# Diagnostic indicators of non-cardiovascular chest pain: a systematic review and meta-analysis

**DOI:** 10.1186/1741-7015-11-239

**Published:** 2013-11-08

**Authors:** Maria M Wertli, Katrin B Ruchti, Johann Steurer, Ulrike Held

**Affiliations:** 1 Horten Center for Patient Oriented Research and Knowledge Transfer, Department of Internal Medicine, University of Zurich, Pestalozzistrasse 24, CH-8032, Zurich, Switzerland

## Abstract

**Background:**

Non-cardiovascular chest pain (NCCP) has a high healthcare cost, but insufficient guidelines exist for its diagnostic investigation. The objective of the present work was to identify important diagnostic indicators and their accuracy for specific and non-specific conditions underlying NCCP.

**Methods:**

A systematic review and meta-analysis were performed. In May 2012, six databases were searched. Hand and bibliography searches were also conducted. Studies evaluating a diagnostic test against a reference test in patients with NCCP were included. Exclusion criteria were having <30 patients per group, and evaluating diagnostic tests for acute cardiovascular disease. Diagnostic accuracy is given in likelihood ratios (LR): very good (LR+ >10, LR- <0.1); good (LR + 5 to 10, LR- 0.1 to 0.2); fair (LR + 2 to 5, LR- 0.2 to 0.5); or poor (LR + 1 to 2, LR- 0.5 to 1). Joined meta-analysis of the diagnostic test sensitivity and specificity was performed by applying a hierarchical Bayesian model.

**Results:**

Out of 6,316 records, 260 were reviewed in full text, and 28 were included: 20 investigating gastroesophageal reflux disorders (GERD), 3 musculoskeletal chest pain, and 5 psychiatric conditions. Study quality was good in 15 studies and moderate in 13. GERD diagnosis was more likely with typical GERD symptoms (LR + 2.70 and 2.75, LR- 0.42 and 0.78) than atypical GERD symptoms (LR + 0.49, LR- 2.71). GERD was also more likely with a positive response to a proton pump inhibitor (PPI) test (LR + 5.48, 7.13, and 8.56; LR- 0.24, 0.25, and 0.28); the posterior mean sensitivity and specificity of six studies were 0.89 (95% credible interval, 0.28 to 1) and 0.88 (95% credible interval, 0.26 to 1), respectively. Panic and anxiety screening scores can identify individuals requiring further testing for anxiety or panic disorders. Clinical findings in musculoskeletal pain either had a fair to moderate LR + and a poor LR- or vice versa.

**Conclusions:**

In patients with NCCP, thorough clinical evaluation of the patient’s history, symptoms, and clinical findings can indicate the most appropriate diagnostic tests. Treatment response to high-dose PPI treatment provides important information regarding GERD, and should be considered early. Panic and anxiety disorders are often undiagnosed and should be considered in the differential diagnosis of chest pain.

## Background

In the USA, 6 million patients present to emergency departments with chest pain each year, at an annual cost of $8 billion [1,2]. In emergency departments, roughly 60% to 90% of the patients presenting with chest pain have no underlying cardiovascular disease [3-6]. The proportion of patients with cardiovascular disease may be higher in specialized units (cardiology emergency departments, cardiac care units (CCUs), intensive care units (ICUs)) [7] and lower in the primary care setting [6,8-10]. Physicians generally assume that patients with non-cardiovascular chest pain (NCCP) have an excellent prognosis after ruling out serious diseases. However, patients with NCCP have a high disease burden; most patients that seek care for NCCP complain of persisting symptoms on 4-year follow-up [11]. Furthermore, compared to patients with cardiac pain, patients with non-cardiac chest pain have a similarly impaired quality of life and similar numbers of doctor visits [12].

In patients with chest pain, the diagnostic investigation focuses primarily on cardiovascular disease diagnosis and is often performed by cardiologists. Upon ruling out cardiovascular disease, only vague recommendations exist for further diagnostic investigation, often delaying diagnosis and appropriate treatment and causing uncertainty for patients [13]. Limited data are available regarding efficient diagnostic investigations for patients with NCCP. Most studies investigate gastrointestinal diseases, and extensive provocation testing has been proposed [14]. Some report that almost half of the patients with NCCP will have gastrointestinal disorders [12], while others attribute more than a third of cases to psychiatric disorders, as diagnosed by the *Diagnostic and Statistical Manual of Mental Disorders*, fourth edition (DSMIV). Referred pain from the spine and the chest wall are also likely substantial contributors to NCCP. Information is scarce regarding the appropriate diagnostic tests, and their sensitivity and specificity to discriminate different non-cardiac diseases.

The present systematic review aimed to identify relevant diagnostic tests for patients with NCCP, and to summarize their positive and negative likelihood ratios for underlying disease identification.

## Methods

### Literature search and study selection

This review, conducted in May 2012, followed the QUADAS quality assessment checklist for diagnostic accuracy studies [15]. We searched six databases (PubMed/Medline, Biosis/Biological Abstracts (Web of Knowledge), Embase (OvidSP), INSPEC (Web of Knowledge), PsycInfo (OvidSP), and Web of Science (Web of Knowledge)) using the following search terms as medical subject headings (MeSH) and other subject headings: thoracic pain, chest pain, non-cardiac chest pain, atypical chest pain, musculoskeletal chest pain, esophageal chest, and thoracic spine pain. The findings were limited to studies investigating ‘diagnosis’, ‘sensitivity and specificity’, ‘sensitivity specificity’, or chest pain/diagnosis. We applied no limits for study setting or language; however, one potentially eligible Russian language article was excluded due to lack of language proficiency [16]. Appendix 1 depicts three detailed search strategies.

To ensure search completeness, one reviewer (MW) conducted a hand search of the last 5 years in the four journals that published most articles about patients with NCCP (*Gastroenterology*, *Chest*, *Journal of the American College of Cardiology* and *American Journal of Cardiology*). Potentially eligible references not retrieved by the systematic search in the six databases were added. Bibliographies of included studies were also searched, and potential eligible references included in the full text review.

### Eligibility criteria

Eligible studies included non-screening studies on diagnostic accuracy published between 1992 and May 2012. Inclusion criteria were studies reporting on patients of 18 years and older, seeking care for NCCP. NCCP was defined as chest pain and cardiac or other vascular disease was ruled out (that is, cardiovascular disease, aortic dissection, pulmonary embolism). Exclusion criteria included studies with <30 patients per group due to concerns about sample size [17]. This group size was arbitrarily chosen to exclude studies with the highest risk of bias, while allowing a comprehensive literature overview. Based on the nomogram proposed by Carley *et al*. a sample size of more than >150 patients are needed to accurately assess a diagnostic test [18]; however, with this sample size cut-off, very few studies (mainly retrospective data analyses) would have been eligible.

### Study selection, data extraction, and synthesis

Two reviewers (MW and KR) independently screened 6,380 references by title and abstract. Both reviewers independently reviewed the full text of 260 studies meeting the eligibility criteria. Disagreements were discussed and resolved by consensus or third party arbitration (JS). Researchers with specific language proficiencies reviewed non-English language references. When the same study was included in several publications without change in diagnostic measure, the most recent publication was chosen and missing information was added from previous publications.

All information regarding the diagnostic test, reference test, and considered differential diagnosis was extracted and grouped according to the disease investigated. The methods used to assess accuracy, sensitivity, and specificity were also extracted.

### Quality assessment

Study quality was assessed using the Scottish Intercollegiate Guidelines Network (SIGN) methodology checklist for diagnostic studies [19]. Overall bias risk and study quality was rated according to the SIGN recommendations. The ratings included high quality (++; most criteria fulfilled and if not fulfilled, the study conclusions are very unlikely to be altered), moderate quality (+; some criteria fulfilled and if not fulfilled, the study conclusions are unlikely to be altered), low quality (−; few or no criteria fulfilled, conclusions likely to be altered). Studies rated as low quality by both reviewers were excluded from further analysis.

### Reference standards and test evaluation

Information about method validity, reliability, practicability and value for clinical practice of the reference and the standard test was extracted and critically assessed. When several reference standards were used, all measurements were extracted and used for further analysis.

### Statistical analysis

Descriptive statistics were used to summarize findings across all diagnostic studies. Sensitivity, specificity, positive and negative predictive values (PPV and NPV, respectively), and positive and negative likelihood ratios (LR + and LR-, respectively) were calculated based on a 2 × 2 table (true/false positives, true/false negatives). Pretest probabilities (prevalence) and the positive and negative post-test probability of the disease were calculated. If one field contained the value 0, 0.5 was added to each field to enable value calculation. Test diagnostic accuracy was assessed as follows [20]: very good (LR+ >10, LR- <0.1); good (LR + 5 to 10, LR- 0.1 to 0.2); fair (LR + 2 to 5, LR- 0.2 to 0.5); or poor (LR + 1 to 2, LR- 0.5 to 1).

When more than four unbiased studies were available in clinically similar populations and with comparable index and reference tests, we performed joined meta-analysis of the diagnostic test sensitivity and specificity. We used a hierarchical Bayesian model, as proposed by Rutter and Gatsonis [21], which accounts for the within-study and between-study variability and the potentially imperfect nature of the reference test. The hierarchical Bayesian model was set up as follows: we assumed *J* diagnostic studies in the meta-analysis, with crosstabulation between index test (*T1*) and reference test (*T2*) available for each study, and both tests assumed to be dichotomous (1 = positive test result, 0 = negative test result). Each study was assumed to use a different cut-off value (θ_j_) to define a positive test result. The diagnostic accuracy of each study was denoted by α_j_. The model structure implied a within-study level for study-specific parameters (θ_j_ and α_j_), and a between-study level for parameters common among all studies. The model could theoretically be extended to include study-specific covariates such as percentage of female patients or mean age to reduce heterogeneity on study level.

Appendix 2 gives details of the model set up and prior distributions. The results of the Bayesian analysis are samples from the posterior distribution of the parameters, and estimates are presented as posterior means (50% quantile), and lower (2.5% quantile) and upper (97.5% quantile) bounds, resulting in a 95% credible region. Analyses were performed using R statistical software and the ‘HSROC’ package [22,23].

### Ethics statement

For this study no ethical approval was required. No protocol was published or registered. All methods were determined a priori.

## Results

### Study selection

**Figure 1:**
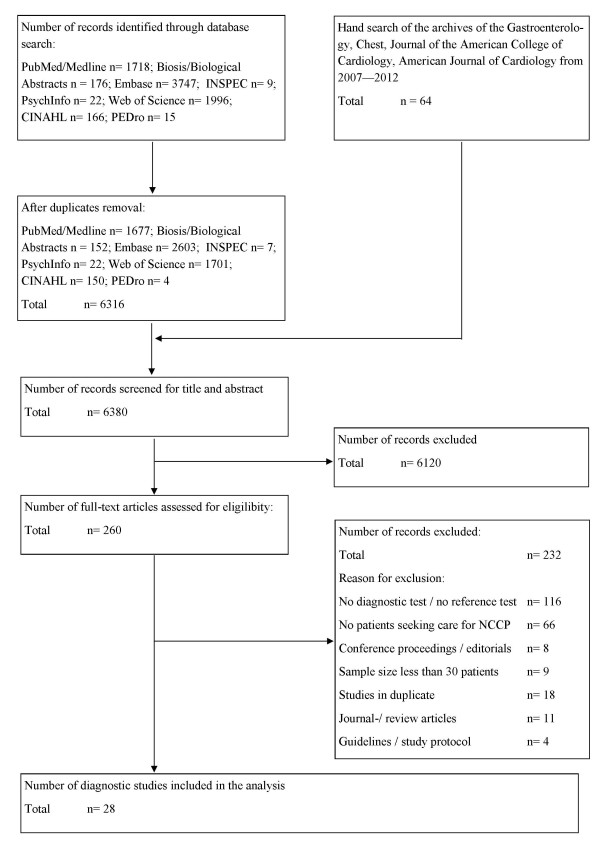
Study flow.

Figure 1 summarizes the search and inclusion process. Out of 6380 records, 260 were reviewed in full text, resulting in exclusion of 232 studies. In total, the analysis included 28 studies. The reasons for exclusion of the 232 studies are given in Figure 1 and overview of excluded studies reviewed in full text is give in Appendix 3.

### Study characteristics

Table 1 presents the study characteristics, and included patients. In all, 20 studies (71%) evaluated diagnostic tests to identify gastrointestinal disease, mainly gastroesophageal reflux disorders (GERD), underlying NCCP. Musculoskeletal chest pain was investigated in three studies (11%), and psychiatric conditions in five studies (18%). Study quality was good in 15 studies (54%) and moderate in 13 (46%; Appendix 4). No study had to be excluded because of poor study quality.

**Table 1:** Baseline characteristic of the studies

**Author**	**Study design**	**Recruitment**	**Inclusion criteria**	**Exclusion criteria**	**n all (n female), subgroups**	**Age, mean (SD)**	**Disease duration**
Symptoms suggesting gastroesophageal reflux (GERD)-related non-cardiac chest pain (NCCP)							
Kim *et al*., 2007 [24]	Cross-sectional, funding NR	Inpatients with NCCP, referred by a cardiologist after negative cardiac evaluation. Tertiary care, Seoul, Korea.	NCCP was defined when patients were admitted for chest pain to the coronary unit for ≥1 episode of unexplained chest pain/week for ≥3 months. Cardiac chest pain was ruled out by electrocardiogram (ECG), normal enzymes, negative treadmill exercise testing, normal or insignificant ECG changes after intravenous ergonovine injection in coronary angiograms.	Severe liver, lung, renal or hematological disorders. History of peptic ulcer or gastrointestinal (GI) surgery, connective tissue disorder and chest pain originating in a musculoskeletal disorder.	58 (female 37), NCCP with GERD symptoms (sy) 24, NCCP without GERD sy 34	54.6 (10.4)	17% <6 months, 17% 6 to 12 months, 51% 1 to 5 years, 16% >5 years
Hong *et al*., 2005 [25]	Retrospective data analysis, funding NR	Patients with a clinical suspicion of esophageal motility abnormalities and pathological acid exposure within 1 month were included in this analysis. Tertiary care, Seoul, Korea.	Patients with suspicion of esophageal motility abnormalities and pathological acid exposure. NCCP was defined as recurrent angina-like or substernal chest pain believed to be unrelated to the heart, after comprehensive evaluation by the cardiologist.	Obstructive lesions, previous esophageal balloon dilatation, botulism toxin injection, or anti-reflux surgery. No complaints associated with symptoms centered on the esophagus. Connective tissue diseases.	462 (female 269), dysphagia 53, NCCP 186, GERD sy 117	47.6 (10.9)	NR
Netzer *et al*., 1999 [26]	Retrospective data analysis, funding NR	First-time referrals to esophageal function testing laboratory. Tertiary care, Bern, Switzerland.	First-time referrals to esophageal function testing laboratory. NCCP group included all patients referred for GI testing because of NCCP. Additional information was obtained by contacting the general practitioner (GP) and interviews.	NR	303 (female 145), GERD 143, dysphagia 56, NCCP 45	50 (15)	NR
Mousavi *et al*., 2007 [27]	Prospective observational, funding NR	Outpatient referral by cardiologist after non-invasive diagnostic evaluation and exclusion of a cardiac or other source. Semnan, Iran.	Patients with NCCP referred to the gastrointestinal clinic. NCCP was diagnosed when chest pain was believed to be unrelated to the heart after an evaluation by a cardiologist including non-invasive testing and no apparent other diagnosis was present.	Non-steroidal anti-inflammatory drug (NSAID) use, peptic stricture, duodenal/gastric ulcer. History of upper GI surgery, scleroderma, diabetes mellitus, neuropathy, myopathy or functional bowel disorders, any condition that may affect lower esophageal sphincter pressure or decrease acid clearance time.	78 (female 37), NCCP with GERD sy. 35, NCCP without GERD 43	50.4 (2.3)	3 to 30 days (mean 9.3 ± 4.2 days)
Singh *et al*., 1993 [28]	Retrospective data analysis, funding NR	All consecutive outpatients referred to Esophageal Laboratory for evaluation of upper gastrointestinal complaints. Alabama, USA.	61 patients had NCCP and were analyzed in comparison to reflux patients for findings in upper gastrointestinal endoscopy and ambulatory 24 h pH monitoring	NR	153 (female 40)	NR	NR
Ho *et al*., 1998 [29]	Cross-sectional, research grant, National University of Singapore	Outpatient referral for NCCP to the gastroenterology service. Tertiary care, Singapore.	Recurrent NCCP ≥3 months. Normal cardiac evaluation (non-obstructed coronary arteries (<50% diameter narrowing), dobutamine stress echocardiography, exercise ECG). Cardiologist evaluation not cardiac.	No history of esophageal disorder or esophageal surgery	61 (NR)	NR	≥3 months
Lam *et al*., 1992 [30]	Cross-sectional, funding NR	Patients referred to the gastroenterologist after being released from a cardiac care unit (CCU) where they were admitted with suspected myocardial infarction but negative cardiac evaluation. Secondary care, Haarlem, The Netherlands. Patients were eligible for the study when a cardiologist determined the chest pain to be of non-cardiac origin.	Episode of acute, prolonged retrosternal chest pain. Cardiac chest pain was ruled out when no abnormalities on admission ECG, negative results on heart enzyme tests, negative exercise test. Further cardiac testing (coronary angiography) was only performed when considered necessary by the cardiologist.	Age >80 years, ECG ischemic alterations on the admission, arrhythmias, or signs of congestive heart failure	41 (female 41)	61.4 (range 40 to 75)	Acute episode of chest pain
Studies investigating the efficacy and diagnostic value of proton pump inhibitor (PPI) trials in GERD-related NCCP							
Dickman *et al*., 2005 [31]	Randomized, controlled trial (RCT), double-blind, crossover, Janssen Pharmaceutica und Eisai Inc.	Outpatient referral by a cardiologist after negative cardiac evaluation. Tertiary care, Arizona, USA.	NCCP ≥3 episodes/week (angina-like) for ≥3 months. Normal/insignificant findings coronary angiogram, or insufficient evidence for ischemic heart disease (IHD) in non-invasive tests.	Severe comorbidity, previous empirical anti-reflux regimen, history of peptic ulcer disease or gastrointestinal surgery	35 (female 12), GERD + 16 (45.7%), GERD- 19 (54.3%)	55.6 (10.10)	≥3 months
Bautista *et al*. 2004 [32]	RCT, double-blind, crossover, TAP Pharmaceuticals	Outpatient referral by a cardiologist after negative cardiac evaluation. Tertiary care, Arizona, USA.	NCCP ≥3 episodes (angina-like) for ≥3 months. Normal/insignificant findings coronary angiogram, or insufficient evidence for IHD in non-invasive tests.	Severe comorbidity, previous empirical anti-reflux regimen, history of peptic ulcer disease or gastrointestinal surgery	40 (female 9), placebo 40, GERD + 18, GERD- 22	54.4 (2.78)	≥3 months
Fass *et al*. 1998 [33]	RCT, double-blind, crossover, Astra-Merck research grant	Outpatient referral by a cardiologist after negative cardiac evaluation. Tertiary care, Arizona, USA.	NCCP ≥3 episodes (angina-like) for ≥3 months. Normal/insignificant findings coronary angiogram, or insufficient evidence for IHD in non-invasive tests.	Previous empirical anti-reflux regimen, history of peptic ulcer disease or gastrointestinal surgery	37 (female 1), GERD + 23, GERD- 14	58.2 (2.3)	≥3 months
Pandak *et al*., 2002 [34]	RCT, double-blind, crossover, Astra Zeneca	Patients presented with recurrent chest pain, whose chest pain was determined by cardiologist to be of non-cardiac origin with the aid of methoxyisobutylisonitrile (MIBI) testing. Tertiary care, Arizona, USA.	Unexplained recurrent chest pain determined to be of non-cardiac origin by a cardiologist and had negative results on MIBI testing	Previous empirical anti-reflux regimen, gastric or duodenal ulcer, prior gastric surgery, abnormalities on physical exam or chest x-ray that would explain the chest pain	42 (female 24), GERD + 20, GERD- 18	Range 22 to 77	≥6 months
Kim *et al*., 2009 [35]	Prospective observational, Janssen Pharmaceuticals	Inpatients referred after negative cardiac examination by cardiologists to gastroenterology. Tertiary care, Seoul, Korea.	NCCP was defined when patients were admitted for chest pain to the coronary unit for ≥1 episode of unexplained chest pain/week for ≥3 months. Cardiac chest pain was ruled out by ECG, normal enzymes, negative treadmill exercise testing, normal or insignificant ECG changes after intravenous ergonovine injection in coronary angiograms.	Severe comorbidity, history of peptic ulcer disease or gastrointestinal surgery, history of connective tissue disorder and chest pain originating from musculoskeletal disorder	42 (female 17), GERD + 16, GERD- 26	53.9 (12.8)	≥3 months: n = 12 3 to 12 months; n = 23 1 to 5 years; n = 7 >5 years
Xia *et al*., 2003 [36]	RCT, single blind, Simon KY Lee Gastroenterology Research Fund	Referred by a cardiologist after negative cardiac evaluation. Tertiary care, Hong Kong, China.	NCCP ≥12 weeks during last 12 months. Normal coronary angiograph, chest pain considered by a cardiologist to be NCCP.	Pathologic endoscopic finding, previous anti-reflux regimen, apparent heartburn, acid reflux, dysphagia and dyspepsia	68 (female 42), placebo 32, lansoprazole 36	58.2 (10.0)	≥12 weeks
Kushnir *et al*., 2010 [37]	Retrospective data analysis, Mentors in Medicine, Washington University, St Louis, MO, USA	Outpatients referred for ambulatory pH monitoring for the evaluation of unexplained chest pain. Tertiary care, Missouri, USA.	Unexplained chest pain. Cardiac causes were excluded in all instances before referral.	Anti-reflux surgery in the past, chest pain was not the dominant symptom, pH manometry data incomplete	98 (female 75)	51.8 (1.1)	7.4 ± 4.1 years
Lacima *et al*. 2003 [38]	Cross-sectional, funding NR	Referred by a cardiologist after negative cardiac evaluation. Barcelona, Spain.	Normal ECG, cardiac enzymes, treadmill exercise testing, coronary angiography and epicardial coronary arteries or with <25% narrowing, no ECG changes after intravenous ergonovine injection	Previous anti-reflux regimen, calcium channel blockers, beta blockers and/or nitrates were withdrawn at least 7 days before the study	120 (female 62), patients 90, volunteers 30	57 (27 to 82)	NR
Studies investigating the value of provocation tests for the diagnosis of GERD-related NCCP							
Cooke *et al*., 1994 [39]	Cross-sectional, funding NR	Patients in whom coronary angiography was performed for the diagnosis of new chest pain. Secondary care, London, UK.	New chest pain and normal coronary anatomy with exertional pain as principal complaint	Mitral valve prolapse, left ventricular hypertrophy, previous myocardial infarction, abnormalities of resting wall motion on echocardiography, pain at rest only, unable to exercise. Previous anti-reflux regimen, previous gastroenterologist assessment.	66 (female 34), non-cardiovascular disease (CVD) 50, CVD 16 (controls)	53 (non-CVD), CVD 58, range 32 to 72	3.4 years
Bovero *et al*., 1993 [40]	Cross-sectional, funding NR	Patients investigated for chest pain. Secondary care, Genova, Italy.	Chest pain, no coronaroactive drugs for ≥5 days. No anti-reflux regimen ≥3 days.	Chest pain of organic and/or functional cardiologic origin (evaluated by: ECG, two ergometry tests, dynamic ECG, thallium myocardial scintigraphy under physical stress or echodypiridamole test, ergonovine or methyl-ergometrine test, angiography)	67 (female 43), pain at rest 46, exertional pain 21	53 (range 34 to 76)	NR
Romand *et al*., 1999 [41]	Cross-sectional, funding NR	Referred after negative cardiac evaluation. Secondary care, Lyon, France.	Normal coronary anatomy, normal ECG, negative treadmill exercise	Cardiologic origin of symptoms, history of upper gastrointestinal surgery, duodenal or gastric ulcer, peptic stricture or stricture by a tumor	43 (female 19)	56 (range 31 to 78)	n = 25 <1 year; n = 7 1 to 5 years; n = 11 >5 years
Abrahao *et al*., 2005 [42]	Cross-sectional, funding NR	Referred by a cardiologist after negative cardiac evaluation. Tertiary care, Rio de Janeiro, Brazil.	≥1 episode of NCCP/week, normal coronary angiogram or with <30% narrowing	Chronic obstructive lung disease, asthma, cardiac arrhythmia, cardiomyopathy, valvular heart disease	40 (female 32)	54.7 (8.4)	Mean 24 months (range 1 to 360 months)
Ho *et al*., 1998 [29]	Cross-sectional, research grant from the National University of Singapore	Referred for NCCP to the gastroenterology service, Singapore	Recurrent chest pain of ≥3 months; cardiologists evaluation normal and symptoms not cardiac (non-obstructed coronary arteries (<50% luminal narrowing), dobutamine stress echocardiography, exercise ECG)	No history of proven esophageal disorder or esophageal surgery	80 (female 38)	48 (range 21 to 75)	≥3 months
Eosinophilic esophagitis-related NCCP							
Achem *et al*., 2011 [43]	Retrospective data analysis, funding NR	Referred for endoscopic evaluation of NCCP, who had esophageal biopsies for suspected eosinophilic esophagitis. Secondary care, Florida, USA.	Chest pain suspected of being esophageal origin after negative cardiac evaluation (either by non-invasive stress testing or coronary angiography)	Dysphagia (if this was the main reason for endoscopy). Anticoagulant use.	171 (female 104), 24 (female 7) eosinophilia, 147 (female 97) normal histology	59 (24 to 86) normal histology, 55 (21 to 81) eosinophilia	NR
Musculoskeletal NCCP							
Stochkendahl *et al*., 2012 [44]	Cross-sectional, Foundation Chiropractic Research and Postgraduate Education, Government	Patients discharged form an emergency cardiology department. Tertiary care, Odense, Denmark.	Acute (<7 days) chest pain primary complaint. Pain in the thorax and/or neck. Understand Danish. Age 18 to 75 years, resident of the Funen County.	Cardiovascular disease, previous percutaneous coronary intervention or coronary artery bypass graft: other definite cause, inflammatory joint disease, insulin dependent diabetes, fibromyalgia, malignant disease, apoplexy, dementia or unable to cooperate, major osseous anomaly, osteoporosis, pregnancy	302 (female 132)	52.5 (11.0)	Acute episode, <7 days before admission
Bosner *et al*. 2010 [45]	Cross-sectional with 6 months follow-up, federal Ministry of Education and Research grant	Consecutive recruitment of all patients presenting to chest pain in a GP clinic. An independent interdisciplinary reference panel decided about the etiology of chest pain.	Age >35 years, pain (acute or chronic) localized between clavicles and lower costal margins and anterior to the posterior axillary lines	Patients whose chest pain had been investigated already and/or who came for follow-up for previously diagnosed chest pain were excluded	1,212 (female 678), chest wall symptom (CWS) 565 (female 330)	All 59 (35 to 93), CWS 58 (35 to 90)	Acute pain 28.4%
Manchikanti *et al*., 2002 [46]	Cross-sectional, no funding	Chronic thoracic pain, managed by one physician and undergoing diagnostic medial branch blocks. Private pain practice, USA.	Pain for ≥6 months. Failure of conservative management with physical therapy, chiropractic management and drug therapy. Age 18 to 90 years.	No radicular pattern of pain, no disc herniation on MRI	46 (female 31)	46 (2.2)	≥6 months, mean 86 (SD 17.2) months
NCCP related to psychiatric diseases							
Kuijpers *et al*., 2003 [47]	Cross-sectional, funding NR	Discharged from the hospitals first-heart-aid service with a diagnosis of NCCP received an envelope	Chest pain or palpitation presenting to first-heart-aid service, received no cardiac explanation	Dementia, live ≥50 km from the hospital. Do not speak Dutch.	344 (female 151), Hospital Anxiety Depression Scale (HADS) ≥8: 266 (female 123); HADS <8: 78 (female 28)	HADS ≥8: 55.81 (13.03); HADS <8: 60.55 (10.84)	NR
Demiryoguran *et al*., 2006 [48]	Cross-sectional, funding NR	Patients admitted to the ER and discharged with a diagnosis of NCCP. Ismir, Turkey.	Cardiac chest pain ruled out. Normal ECGs and low or stable levels of cardiac markers.	Unstable vital signs, uncooperative and disoriented patients. Established diagnoses. Documented coronary artery disease, history of trauma to chest wall, back or abdomen within the previous week.	157 (female 89), HADS <10: 108 (female 55), HADS >10: 49 (female 34)	41.6 (11.7)	NR
Foldes-Busque *et al*., 2011 [49]	Cross-sectional, Groupe interuniversitaire de recherche sur les urgences (GIRU) and Fonds de Recherche en Santé du Québec	Emergency department (ED), Monday to Friday between 8 AM and 4 PM. Tertiary care, Quebec, Canada.	Low-risk unexplained chest pain, ≥18 years old. English or French speaking, normal serial ECG, normal cardiac enzymes.	Explained chest pain (for example, ischemic, cause identifiable by radiography). Medical condition that could invalidate the interview (for example, psychosis, intoxication, or cognitive deficit), any unstable condition, or any trauma.	507 (NR), derivation sample 201 (female 101); validation sample 306 (female 173)	Derivation condition 54.2 (13.9), validation condition 53.3 (14.4)	NR
Fleet *et al*. 1997 [50]	Cross-sectional, Fonds de Recherché en Santé Québec	Consecutive patients presenting to ambulatory walk in ED, patients with or without IHD, Québec, Canada	Complaint of chest pain, understand French, able to complete evaluation in the ED	Cognitive impairment, psychotic state	Derivation sample 180 (female 63), validation sample 212	Development 57.6 (12.6), validation 56 (12.2)	NR
Katerndahl *et al*., 1997 [51]	Cross-sectional, public health and service Establishment of Departments of Family Practice	Presented to the GP with a chief compliant of new-onset chest pain. Primary care, Texas, USA.	Adults 18 years and older, new-onset chest pain, only one complaint (chest pain) as well as those with several symptoms that included chest pain	Previous investigation for chest pain at the practice	51 (NR)	42.6 (14.6)	New onset

*NR* not reported.

### Accuracy of symptoms for the diagnosis of GERD

Table 2 summarizes the diagnostic accuracy of the diagnostic tests relevant for clinical practice. A comprehensive overview of all evaluated diagnostic tests is provided in Appendix 5. For diagnosis of GERD, the most common reference tests (endoscopy and/or 24-h pH-metry) are reported.

**Table 2:** Summary of diagnostic accuracy of tests used in non-cardiac chest pain

**Author, year**					
	**Evaluated test**	**Reference standard**	**Prevalence,%**	**LR+**	**LR-**
Symptoms					
Kim *et al*. [24]	NCCP with atypical GERD symptoms	Endoscopy (LA classification) and/or 24 h pH-metry (>4%, pH <4	24	0.49	2.71
Kim *et al*. [24]	NCCP with typical GERD symptoms	Same	67	2.75	0.42
Mousavi *et al*. [27]	NCCP with typical GERD symptoms	GERD if two tests positive: endoscopy (Hentzel-Dent), Bernstein test, omeprazole trial	45	2.70	0.78
Mousavi *et al*. [27]	NCCP relieved by antacid	Same	45	0.51	3.51
Mousavi *et al*. [27]	NCCP and heartburn in past history	Same	45	2.15	0.74
Mousavi *et al*. [27]	NCCP and regurgitation in past history	Same	45	2.98	0.61
Hong *et al*. [25]	NCCP	Manometry (Specler 2001 criteria) and/or 24 h pH-metry (>4% pH <4)	43	0.83	1.13
Hong *et al*. [25]	Control: dysphagia	Same	45	1.27	0.97
Hong *et al*. [25]	Control: GERD-typical symptoms	Same	44	1.26	0.93
Netzer *et al*. [26]	NCCP	Manometry and/or 24 h pH-metry (>10.5% pH <4)	84	0.43	1.23
Netzer *et al*. [26]	Control: GERD-typical symptoms	Same	84	1.53	0.74
Netzer *et al*. [26]	Control: dysphagia	Same	84	1.16	0.97
Proton pump inhibitor (PPI) trial					
Dickman *et al*. [31]	Rabeprazole 20 mg twice a day for 1 week SIS ≥50%	Endoscopy (Hentzel-Dent grades) and/or 24 h pH-metry (>4.2% pH <4)	46	7.13	0.28
Dickman *et al*. [31]	Placebo for 1 week	Same	46	0.89	1.03
Bautista *et al*. [32]	Lansoprazole 60 mg AM, 30 mg PM for 1 week SIS ≥50%	Endoscopy (Hentzel-Dent grades) and/or 24 h pH-metry (>4.2% pH <4)	45	8.56	0.24
Bautista *et al*. [32]	Lansoprazole 60 mg AM, 30 mg PM for 1 week SIS ≥65%	Same	45	18.33	0.17
Bautista *et al*. [32]	Placebo for 1 week	Same	45	0.61	1.22
Fass *et al*. [33]	Omeprazole 40 mg AM, 20 mg PM for 1 week SIS ≥50%	Endoscopy (Hentzel-Dent grades) and/or 24 h pH-metry (>4.2% pH <4)	62	5.48	0.25
Fass *et al*. [33]	Placebo for 1 week	Same	62	3.04	0.84
Pandak *et al*. [34]	Omeprazole 40 mg twice a day for 2 weeks SIS ≥50%	Endoscopy and/or 24 h pH-metry (>4.2% pH <4)	53	2.70	0.15
Pandak *et al*. [34]	Placebo for 2 weeks SIS ≥50%	Same	53	0.30	1.14
Kim *et al*. [35]	NCCP rabeprazole for 1 week SIS ≥50%	Endoscopy (LA classification) and/or 24 h pH-metry (>4.0 pH <4)	38	2.17	0.65
Kim *et al*. [35]	NCCP rabeprazole for 2 weeks SIS ≥50%	Same	38	3.02	0.26
Xia *et al*. [36]	Lansoprazole 30 mg once a day for 4 weeks SIS ≥50%	24 h pH-metry (De Meester pH <4, 7.5 s)	33	2.75	0.13
Xia *et al*. [36]	Placebo for 4 weeks SIS ≥50%	Same	38	0.95	1.03
Kushnir *et al*. [37]	High-degree response on PPI (not specified)	24 pH-metry (≥4%, pH <4)	53	1.97	0.38
Provocation test					
Cooke *et al*. [39]	NCCP during exertional pH-metry	24 h pH-metry (5.5% pH <4 for 10 s)	38	14.40	0.79
Cooke *et al*. [39]	Control group: CVD with angina: exertional pH-metry	Same	19	4.33	0.72
Bovero *et al*. [40]	NCCP with normal ECG during exertional pH-metry	24 h pH-metry (De Meester criteria: >4.5% pH <4))	69	7.76	0.66
Bovero *et al*. [40]	NCCP at rest: NCCP with normal ECG during exertional pH-metry	Same	74	3.88	0.74
Bovero *et al*. [40]	NCCP exertion/mixed: NCCP with normal ECG during exertional pH-metry	Same	57	10.00	0.50
Romand *et al*. [41]	NCCP: pH <4 for 10 s during exertional pH-metry	24 h pH-metry (De Meester criteria: >4.5% pH <4))	23	1.65	0.52
Abrahao *et al*. [42]	NCCP reproducible during balloon distension	Endoscopy (Savary-Miller) and/or manometry and/or pH-metry (De Meester criteria: >4.5% pH <4	88	2.00	0.75
Abrahao *et al*. [42]	NCCP reproducible during Tensilon test	Same	88	0.43	1.38
Abrahao *et al*. [42]	NCCP reproducible during Bernstein test	Same	88	1.29	0.93
Abrahao *et al*. [42]	Tensilon and Bernstein Test and balloon distension (+ if 1 test +)	Same	88	0.95	1.07
Ho *et al*. [29]	NCCP reproducible during Bernstein test	24 h pH-metry (>4% pH <4, 4 s)	23	0.75	1.06
Musculoskeletal disorders					
Stochkendahl *et al*. [44]	≥3 of 5 palpation findings: (1) sitting motion of end-play restriction in lateral flexion and rotation segment C4 to C7 and Th1 to Th8. (2) Prone motion joint-play restriction segment Th1 to Th8. (3) Prone evaluation paraspinal tenderness segment Th1 to Th8. (4) Supine manual palpation muscular tenderness of 14 points anterior chest wall. 5) Supine evaluation of tenderness of the costosternal junctions of costa 2 to 6 and xiphoid process	Diagnosis using a standardized examination protocol:	37	1.52	0.03
		(1) A semistructured interview: pain characteristics, lung and gastrointestinal symptoms, past medical history, height, weight, cardiovascular risk factors			
		(2) A general health examination: blood pressure, pulse, heart and lung stethoscopy, abdominal palpation, neck auscultation, signs of left ventricular failure, neurological examination			
		(3) Manual examination of the muscles and joints (neck, thoracic spine and thorax): active range of motion, manual palpation 14 points muscular tenderness of the anterior chest wall and segmental paraspinal muscles, motion palpation for joint-play restriction of the thoracic spine (Th1 to 8), and end play restriction of the cervical and thoracic spine			
Bosner *et al*. [45]	Chest wall symptom (CWS) score: localized muscle tension, stinging pain, pain reproducible by palpation, absence of cough	Interdisciplinary consensus: cardiologist, GP, research associate (based on reviewed baseline, follow-up data at 6 weeks and 6 months)	47	1.82	0.20
	Cut-off test negative 0 to 1 points				
Bosner *et al*. [45]	CWS score: localized muscle tension, stinging pain, pain reproducible by palpation, absence of cough	Interdisciplinary consensus	47	3.02	0.47
	Cut-off test negative 0 to 2 points				
Stochkendahl *et al*. [44]	Biomechanical dysfunction (part of the standardized examination protocol)^a^	Standardized examination protocol	37	1.58	0.00
Stochkendahl *et al*. [44]	Anterior chest wall tenderness	Standardized examination protocol	37	1.39	0.06
Stochkendahl *et al*. [44]	Angina pectoris (uncertain or negative)	Standardized examination protocol	37	1.26	0.12
Stochkendahl *et al*. [44]	Pain worse on movement of torso	Standardized examination protocol	37	3.39	0.78
Bosner *et al*. [45]	Pain worse with movement	Interdisciplinary consensus	47	2.13	0.75
Stochkendahl *et al*. [44]	Positive/possible belief in pain origin from muscle/joints	Standardized examination protocol	37	1.17	0.20
Stochkendahl *et al*. [44]	Pain relief on pain medication	Standardized examination protocol	37	3.26	0.83
Bosner *et al*. [45]	Pain reproducible by palpation	Interdisciplinary consensus	47	2.08	0.54
Stochkendahl *et al*. [44]	Paraspinal tenderness	Standardized examination protocol	37	1.36	0.48
Bosner *et al*. [45]	Localized muscle tension	Interdisciplinary consensus	47	2.41	0.52
Stochkendahl *et al*. [44]	Chest pain present now	Standardized examination protocol	37	1.35	0.46
Bosner *et al*. [45]	Pain now	Interdisciplinary consensus	47	1.15	0.85
Stochkendahl *et al*. [44]	Pain debut not during a meal	Standardized examination protocol	37	1.10	0.23
Stochkendahl *et al*. [44]	Sharp pain	Standardized examination protocol	37	1.89	0.80
Bosner *et al*. [45]	Stinging pain	Interdisciplinary consensus	47	1.87	0.66
Stochkendahl *et al*. [44]	Hard physical exercise at least once a week	Standardized examination protocol	37	1.19	0.91
Stochkendahl *et al*. [44]	Pain not provoked during a meal	Standardized examination protocol	37	1.09	0.25
Stochkendahl *et al*. [44]	Not sudden debut	Standardized examination protocol	37	2.90	0.63
Bosner *et al*. [45]	Pain >24 h	Interdisciplinary consensus	47	1.30	0.92
Stochkendahl *et al*. [44]	Age ≤49 years old	Standardized examination protocol	37	2.10	0.56
Bosner *et al*. [45]	Pain mostly at noon time	Interdisciplinary consensus	47	0.50	1.02
Bosner *et al*. [45]	Cough	Interdisciplinary consensus	47	0.28	1.18
Bosner *et al*. [45]	Known IHD	Interdisciplinary consensus	47	0.52	1.11
Bosner *et al*. [45]	Pain worse with breathing	Interdisciplinary consensus	47	1.28	0.93
Psychiatric diseases					
Kuijpers *et al*. [47]	Anxiety subscale of the Hospital Anxiety and Depression Scale (HADS-A score, cut-off ≥8)	Diagnosis anxiety disorders (Mini International Neuropsychiatric Interview (gold standard))	58	2.03	0.03
Demiryoguran *et al*. [48]	Chills or hot flushes	Anxiety disorder: HADS-A score (cut-off ≥10)	31	4.85	0.81
Demiryoguran *et al*. [48]	Fear of dying	Anxiety disorder: HADS-A score (cut-off ≥10)	31	4.04	0.82
Demiryoguran *et al*. [48]	Diaphoresis	Anxiety disorder: HADS-A score (cut-off ≥10)	31	3.49	0.69
Demiryoguran *et al*. [48]	Light-headedness, dizziness, faintness	Anxiety disorder: HADS-A score (cut-off ≥10)	31	3.03	0.84
Demiryoguran *et al*. [48]	Palpitation	Anxiety disorder: HADS-A score (cut-off ≥10)	31	1.54	0.83
Demiryoguran *et al*. [48]	Shortness of breath	Anxiety disorder: HADS-A score (cut-off ≥10)	31	1.30	0.92
Demiryoguran *et al*. [48]	Nausea or gastric discomfort	Anxiety disorder: HADS-A score (cut-off ≥10)	31	1.98	0.90
Foldes-Busque *et al*. [49]	The Panic Screening Score (derivation population); does the patient have a history of anxiety disorders? Please indicate how often this thought occurs when you are nervous: ‘I will choke to death’. Did the patient arrive in the ED by ambulance? Please answer the statement by circling the number that best applies to you: ‘When I notice my heart beating rapidly, I worry that I might be having a heart attack’. Sum score 22, A total score ≥6 indicates probable panic.	Panic disorder Diagnosis (structured Anxiety Disorders Interview Schedule for *Diagnostic and Statistical Manual of Mental Disorders*, fourth edition (DSM-IV) (ADIS-IV))	42	3.89	0.44
Foldes-Busque *et al*. [49]	The Panic Screening Score (validation population).	Panic disorder diagnosis (structured ADIS-IV)	43	3.44	0.55
Fleet *et al*. [50]	Panic disorder diagnosis: formula including Agoraphobia Cognitions QA, Mobility Inventory for Agoraphobia, Zone 12 Dermatome Pain Map, Sensory McGill Pain QA, Gender, Zone 25 (validation population)	Panic disorder (ADIS-R structured interview by psychologist)	23	2.60	0.46
Katerndahl *et al*. [51]	GP diagnosis of panic disorder	Panic disorder (structured clinical interview of *Diagnostic and Statistical Manual of Mental Disorders*, based on DSM-III-R)	55	0.82	1.02

LR+: >10; LR-: <0.1; good: LR + 5 to 10, LR- 0.1 to 0.2; fair: LR + 2 to 5, LR- 0.2 to 0.5; poor: LR + 1 to 2, LR- 0.5 to 1.

^a^Biomechanical dysfunction defined as chest pain presumably caused by mechanical joint and muscle dysfunction related to C4 to Th8 somatic structures of the spine and chest wall established by means of joint-play and/or end-play palpation.

Reference tests are as follows. Endoscopic classification: LA classification: grade A, ≥1 mucosal break ≤5 mm, that does not extend between the tops of two mucosal folds; grade B, ≥1 mucosal break >5 mm long that does not extend between the tops of two mucosal folds; grade C, ≥1 mucosal break that is continuous between the tops of two or more mucosal folds but which involves <75% of the circumference; grade D, ≥1 mucosal break which involves at least 75% of the esophageal circumference [52]. Savary-Miller system: grade I, single or isolated erosive lesion(s) affecting only one longitudinal fold; grade II multiple erosive lesions, non-circumferential, affecting more than one longitudinal fold, with or without confluence; grade III, circumferential erosive lesions; grade IV, chronic lesions: ulcer(s), stricture(s) and/or short esophagus. Alone or associated with lesions of grades 1 to 3; grade V, columnar epithelium in continuity with the Z line, non-circular, star-shaped, or circumferential. Alone or associated with lesions of grades 1 to 4 [53]. Hentzel-Dent grades: grade 0, no mucosal abnormalities; grade 1, no macroscopic lesions but erythema, hyperemia, or mucosal friability; grade 2, superficial erosions involving <10% of mucosal surface of the last 5 cm of esophageal squamous mucosa; grade 3, superficial erosions or ulceration involving 10% to 50% of the mucosal surface of the last 5 cm of esophageal squamous mucosa; grade 4, deep peptide ulceration anywhere in the esophagus or confluent erosion of >50% of the mucosal surface of the last 5 cm of esophageal squamous mucosa [54]. pH-metry: De Meester criteria: (1) total number of reflux episodes; (2) number of reflux episodes with pH <4 for more than 5 minutes; (3) duration of the longest episode; (4) percentage total time pH <4; (5) percentage upright time pH <4; and (6) percentage recumbent time pH <4. [55]. Manometry: Spechler criteria is diagnosis of ineffective esophageal motility, nutcracker esophagus, spasm, achalasia based on basal lower esophageal sphincter pressure, relaxation, wave progression, distal wave amplitude [56].

*24-h pH-metry* 24-h pH monitoring, *GERD* gastroesophageal reflux disease, *GP* general practitioner, *IHD* ischemic heart disease, *QA* questionnaire, *Sensory McGill* McGill Pain Questionnaire sensory subscale, *SIS* symptom index score calculated by adding the reported daily severity (mild = 1; moderate = 2; severe = 3; and disabling = 4) multiplied by the reported daily frequency values during each week).

Patients with the main complaint of NCCP were less likely to have GERD (LR + 0.83, 0.43; LR- 1.13, 1.23) compared to patients with the main complaint of dysphagia (LR + 1.27, 1.16; LR- 0.97, 0.97) or GERD typical symptoms without chest pain (LR + 1.26, 1.53; LR- 0.93, 0.74) in two studies [25,26]. Two further studies compared the accuracy of NCCP and typical GERD symptoms (LR + 2.70 [27], 2.75 [24]; LR- 0.42 [24], 0.78 [27]) with NCCP without GERD symptoms (LR + 0.49; LR- 2.71 [24]) or with NCCP and a history of heart burn (LR + 2.15; LR- 0.74 [27]).

### Accuracy of response to proton pump inhibitor (PPI) treatment for diagnosis of GERD in NCCP

The effect of treatment with PPI was measured by using a symptom intensity score (SIS) at baseline and follow-up. The SIS was calculated by adding the reported daily severity (mild = 1; moderate = 2; severe = 3; and disabling = 4) multiplied by the reported daily frequency values obtained during each week of symptom recording.

Table 2 summarizes the results. Three studies compared the treatment response after high doses of PPI (rabeprazole [31], lansoprazole [32], omeprazole [33]) for 1 week to placebo. A reduction of the SIS score of ≥50% was associated with a good LR + and a fair LR- (LR + 5.48 [33], 7.13 [31], 8.56 [32]; LR- 0.24 [32], 0.25 [33], 0.28 [31]) for the presence or absence of GERD. The likelihood ratios in the placebo groups with a reduction of the SIS score of ≥50% were: LR + 0.89 [31], 0.61 [32], 3.04 [33]; LR- 1.03 [31], 1.22 [32], 0.84 [33]. A reduction of the SIS score of ≥65% resulted in a very good LR + (18.33), and a good LR- (0.17) [32]. A treatment duration of 4 weeks (lasoprazole) resulted in a better LR- (LR + 2.75; LR- 0.13) [36] when compared to 2 weeks (omeprazole [34], LR + 2.7; LR- 0.15).

**Figure 2:**
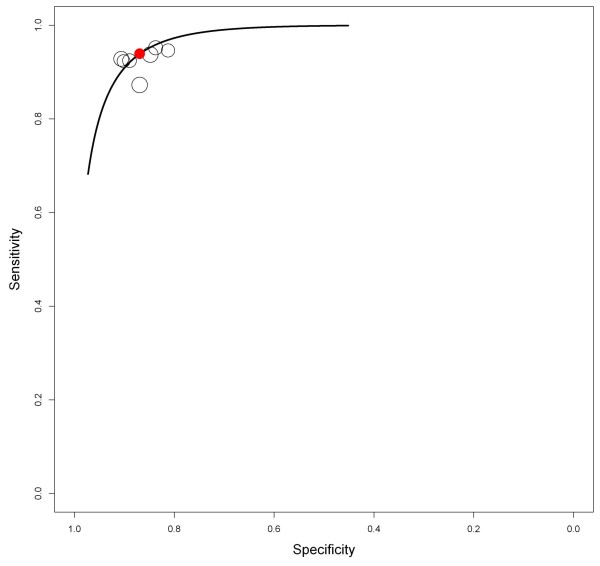
Summary receiver operating characteristic (ROC) curve of proton pump inhibitor (PPI) studies.

For joint meta-analysis only studies were considered with similar study design. Therefore, the active treatment arms of six studies were available for further analysis [31-36]. The model could be extended to include study-specific covariates such as the percentage of female patients or mean age to reduce unexplained heterogeneity on study level. However, due to the small number of studies available for pooling we refrained from including covariates. Figure 2 shows the summary receiver operating characteristic (ROC) curve. Considering the GERD prevalence and the fact that no perfect reference test is available for GERD (sensitivity of the 24-h pH-metry in endoscopy-negative patients <71% [57]), the posterior mean sensitivity of what was 0.89 (95% credible interval, 0.28 to 1). The posterior mean of the specificity was 0.88 (95% credible interval, 0.26 to 1), respectively.

### Accuracy of provocation tests for GERD diagnosis

Using a treadmill test during the 24-h pH-metry (reference test) showed highest LR + for GERD when chest pain was provoked by exercise (LR + 14.4; LR- 0.79 [39]). In all patients who underwent treadmill test, a high number of false negative test results during the treadmill test were observed.

**Figure 3:**
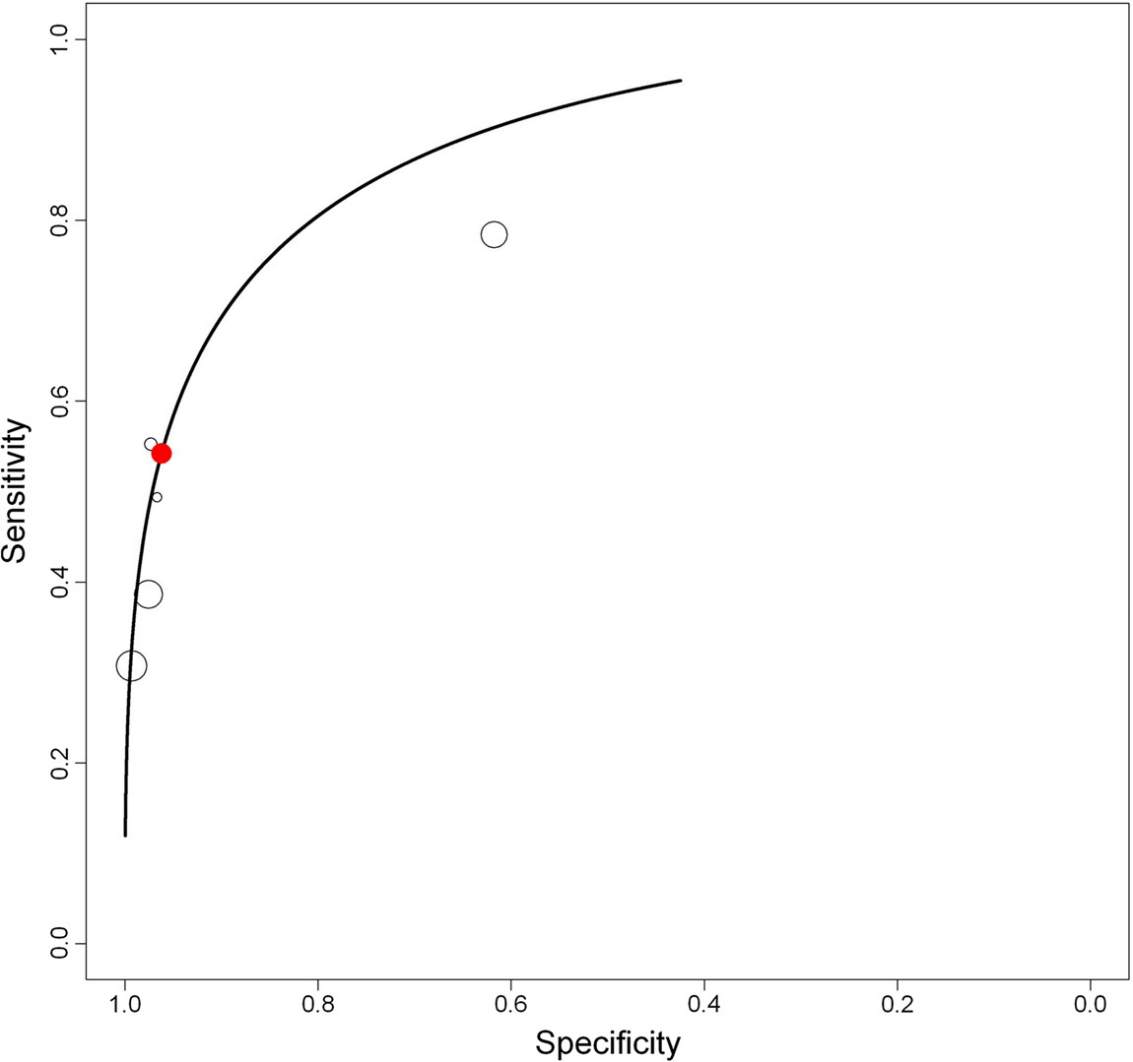
Summary receiver operating characteristic (ROC) for treadmill test during 24 h pH-metry.

For joint meta-analysis only studies were considered with similar study design, again. Five patient groups from four original studies were included in the analysis [38-41]. Figure 3 shows the summary ROC curve. Considering the prevalence and imperfect nature of the reference test, posterior mean sensitivity and specificity were 0.53 (95% credible interval, 0.02 to 1) and 0.93 (95% credible interval, 0.25 to 1), respectively. For all provocation tests (Tensilon test, Bernstein test, or balloon distension test) high numbers of false negative results were found [29,42].

### Accuracy of patient characteristics for eosinophilic esophagitis diagnosis

Eosinophilic esophagitis is a rare but important differential diagnosis for NCCP. In a retrospective analysis the likelihood for histologically proven eosinophilic esophagitis (reference test) was fair when current GERD symptoms were present (LR + 2.36, LR- 0.71 (poor)). Male gender or the presence of typical endoscopic findings for eosinophilic esophagitis were associated with a poor LR + (1.78) but a very good LR- (0.09) No information was available about eosinophilia that responds to PPI treatment compared to eosinophilic esophagitis.

### Accuracy of clinical signs for musculoskeletal chest pain diagnosis

In one study in a cardiology emergency department specific clinical signs or symptoms compared to a standardized examination protocol showed either fair LR + and poor LR- (for example, pain worse with movement of the torso, pain relief on pain medication, no sudden pain start, age ≤49 years) or a poor LR + and a very good LR- (for example, anterior chest wall tenderness, biomechanical dysfunction) [44]. A score of 3 or more points in a sum score (1 point for each of five palpation findings: restriction in C4 to 7/Th1 to Th8 when sitting; prone restriction Th1 to 8; paraspinal tenderness; anterior chest wall tenderness; costosternal junction tenderness) showed an LR + of 1.52 and very good LR- of 0.03. A score of 1 or more points in a sum score for the diagnosis of a chest wall syndrome (CWS) in the GP setting (1 point for each positive finding: localized muscle tension; stinging pain; pain reproducible by palpation; absence of cough) showed a LR + of 1.82 and LR- of 0.20 [45]. A score of 2 or more points in the sum score showed a LR + of 3.02 and LR- of 0.47.

### Accuracy of patient characteristics for psychiatric disease diagnosis

For the diagnosis of an anxiety disorder the anxiety subscale of the Hospital Anxiety and Depression Score (HADS-A, cut-off ≥8) compared to a neuropsychiatric interview (reference test) showed a very good LR- (0.03) and a fair LR + (2.03). In further studies the HADS-A was used as reference test for the diagnosis of anxiety disorder. Specific symptoms showed a fair LR + and a poor LR-: fear of dying (LR + 4.04; LR- 0.82); light-headedness, dizziness, or faintness (LR + 3.03; LR- 0.84); diaphoresis (LR + 3.49; LR- 0.69); and chills or hot flushes (LR + 4.85; LR- 0.81).

For panic disorders a four-item panic screening score validated in patients presenting to an ER showed fair LR + (3.44 and 3.89) and poor-to-fair LR- (0.44 and 0.55) [49]. A combination of different questionnaires and pain patterns (Agoraphobia Cognitions Questionnaire; Mobility Inventory for Agoraphobia; McGill Pain Questionnaire sensory) showed a fair LR + (2.6) and fair LR- (0.46) [50]. In patients presenting to their primary care physician with NCCP the presence of a panic disorder was rarely diagnosed. Clinician consultations in this setting had poor accuracy for panic disorder diagnosis (LR + 0.8; LR- 1.02) [51].

## Discussion

### Main findings

The included studies showed that most studies investigated tests for gastroesophageal reflux disease (GERD) as the underlying disease in non-cardiovascular chest pain (NCCP). Few studies investigated diagnostic tests for other illnesses. The diagnostic value of a PPI treatment test was confirmed, with a ≥50% symptom reduction under PPI treatment showing posterior sensitivity and specificity of almost 90%. Together with the favorable adverse effect profile of PPIs, a high dose (double reference dose, twice daily) can quickly provide important diagnostic information in patients with unexplained chest pain. History or presence of typical GERD-associated symptoms increases the likelihood of GERD.

Only limited evidence was available for other prevalent illnesses manifesting with chest pain. Screening tools for panic and anxiety disorders are valuable for identifying patients requiring further diagnostic evaluation. The likelihood for musculoskeletal chest pain increased when the pain was reproducible or relieved by pain medication. Among studies investigating musculoskeletal disease, the major limitation was the lack of a reference test (‘gold standard’).

### Results in light of existing literature

To the best of our knowledge, this is the first systematic review summarizing the current evidence on the accuracy of diagnostic tests in patients with NCCP. Several non-systematic reviews have suggested various diagnostic and therapeutic approaches [14,58-61], often with algorithms focused on gastrointestinal diseases [14,58,59], sometimes recommending extensive testing, such as provocation tests. Here, we found no additional value of provocation testing for diagnosing underlying gastroesophageal conditions, as provocation tests failed to identify many patients that would have reflux during a 24-h pH measurement period. While meta-analyses of PPI treatment studies compared to placebo have been previously conducted [62,63], compared to this analyses we excluded studies of poor quality and small sample sizes [64-67]. Our study is the first to assess study quality and to use a hierarchical Bayesian approach that accounts for within-study and between-study variability and the imperfect nature of the reference test.

Cremonini *et al*. [62] previously used a bivariate model, and found a lower pooled sensitivity and specificity (sensitivity 80% vs 89%, specificity 74% vs 88%) of a positive PPI treatment response for the diagnosis of GERD. Harbord *et al*. [68] showed that the likelihood functions of the two model formulations are algebraically identical in the absence of covariates. However, for assessing a summary ROC curve, the hierarchical Bayesian model is more natural than the model for pooled sensitivity and specificity [69]. Without a broadly accepted standard reference test, it is important to adjust for conditional dependence between multiple tests (index test and reference test) carried out in the same subjects [69]. The hierarchical Bayesian model can be adapted to this situation by introducing covariance terms between the sensitivities and specificities of the index and reference tests. A previous simulation study [69] demonstrated that if a model does not address an imperfect reference test, bias will be around 0.15 in overall sensitivity and specificity [69]. No systematic review has examined diagnostic studies of musculoskeletal chest pain or chest pain as part of a psychiatric disease.

### Strengths and limitations

This review comprehensively evaluates the currently available studies. The search was inclusive, no language restrictions were applied, and a thorough bibliographic search was conducted to identify all relevant studies. The extraction process was performed in accordance with current guidelines and supported by an experienced statistician. Potential factors influencing diagnostic test accuracy were identified by a multidisciplinary team (an internist, general practitioner, statistician, and methodologist).

The study was limited by the small number of studies available for most diseases presenting with NCCP. Furthermore, many studies were only of moderate quality and most cross-sectional or prospective studies did not meet the required sample size criterion for reliable estimates of sensitivity and specificity. Small studies on diagnostic accuracy are often imprecise, with wide confidence intervals, making it difficult to assess test informativeness [17]. The lack of a gold standard reference test is another limitation, which we addressed within the Bayesian model formulation; however, the resulting posterior credible intervals for overall sensitivity and specificity of the index test are wider than they would be with a perfect reference test. Further, NCCP is a collective term with potentially different underlying diseases and therefore might present differently. Diagnostic accuracy in one population with high prevalence for one disease is high might be entirely different for another population [70]. Therefore, for most studies no joint meta-analysis could be conducted and results have to be interpreted on a single study level within the context of the study population. We have tried to balance this by providing a thorough description of the studies’ inclusion and exclusion criteria and the study setting. This will allow readers to judge to whom study results apply. In studies included in the joint meta-analyses, we intended to include study-specific covariates such as the percentage of female or mean age into the Bayesian model. The inclusion of covariates can reduce unexplained heterogeneity. However, this was due to the small number of studies available for meta-analysis not feasible.

### Research implications

Further research should investigate the combined value of symptoms, clinical findings, and diagnostic tests, including multidisciplinary research aimed at increasing our knowledge about diagnostic processes and making recommendations for diagnostic tests and treatments in patients with NCCP. Most patients with chest pain consult primary care physicians [45], but few studies are performed in this setting. Further research is needed to strengthen the evidence in a primary care setting. The value of screening questionnaires for panic and anxiety disorders should be further evaluated and investigated in clinical practice. The use of a flag system [61], as successfully applied in back and neck pain, could facilitate the diagnostic process allowing systematic assessment of first red flags (acute disease requiring immediate diagnosis and care), then green flags (identifiable diseases), and yellow flags (psychological diseases).

### Implication for practice

Patients with NCCP incur high healthcare costs due to the extensive and often invasive diagnostic testing, and NCCP’s impact on quality of life. Early identification of underlying diseases is essential to avoid delayed treatment and chronicity of complaints. Symptoms and clinical findings may provide important information to guide treatment of an underlying illness. In patients with typical GERD symptoms, twice-daily high-dose PPI treatment is the most efficient diagnostic approach. GERD is very likely if a positive treatment response occurs after 1 week, while GERD is unlikely if there is no response after 4 weeks of PPI treatment. In patients not responding to PPI, if an endoscopy shows no pathological findings, other illnesses should be considered before initiating further gastrointestinal testing.

Panic and anxiety disorders are often missed in clinical practice [51]. Symptoms such as expressing ‘fear of dying’, ‘light headedness, dizziness, faintness’, ‘diaphoresis’ and ‘chills or hot flushes’ are associated with anxiety disorders. Screening tests are valuable to rule out panic or anxiety disorders, and positive finding should lead to further investigation.

## Conclusions

In patients with NCCP, timely diagnostic evaluation and treatment of the underlying disease is important. A thorough history of symptoms and clinical examination findings can inform clinicians which diagnostic tests are most appropriate. Response to high-dose PPI treatment can indicate whether GERD is the underlying disease and should be considered as an early test. Panic and anxiety disorders are often not diagnosed and should be considered in the differential diagnosis of chest pain.

## Appendix 1: Search Strategy May Week 4 2012

In Tables 3 and 4 the detailed search strategy of PubMed, Web of Knowledge (INSPEC, Biosis/Biological Abstracts, Web of Science) and OvidSP (Embase, PsycInfo) are given.

**Table 3:** Search Strategy May Week 4 2012 (PubMed)

**No.**	**Search**	**Hits**
1	Search thoracic pain OR chest pain OR noncardiac chest pain OR non cardiac chest pain OR atypical chest pain OR musculoskeletal chest pain OR esophageal chest pain OR thoracic spine pain OR chest wall	96,313
2	Search coronary artery disease OR cardiac disease OR coronary heart disease OR coronary thrombosis OR coronary occlusion	929,959
3	Search 1 NOT 2	38,735
4	Search sensitivity OR specificity OR diagnostic tests OR chest pain/diagnosis	1,301,595
5	Search 3 AND 4	2,736
6	Search 5 NOT 2; Filters: publication date from 1992/01/01; humans	2,177
7	Search 5 NOT 2; Filters: publication date from 1992/01/01; humans; adult: 19+ years	1,432

### Biological Abstracts/BIOSIS, INSPEC and Web of Science (Web of Knowledge)

Topic = (‘thoracic pain’ OR ‘chest pain’ OR ‘noncardiac chest pain’ OR ‘non cardiac chest pain’ OR ‘atypical chest pain’ OR ‘musculoskeletal chest pain’ OR ‘esophageal chest pain’ OR ‘thoracic spine pain’ OR ‘chest wall’) AND Topic = (sensitivity OR specificity OR diagnostic tests) NOT Topic = (coronary artery disease OR cardiac disease OR coronary heart disease OR coronary thrombosis OR coronary occlusion)

Refined by: Topic = (human*)

Timespan = 1992 to 2012.

**Table 4:** Database: PsycINFO <1806 to May Week 4 2012>, Embase <1974 to 2012 Week 21> (OvidSP)

**No.**	**Search**	**Hits**
1	(thoracic pain or chest pain or noncardiac chest pain or non cardiac chest pain or atypical chest pain or musculoskeletal chest pain or esophageal chest pain or thoracic spine pain or chest wall).mp. [mp = ti, ab, hw, tc, id, ot, tm, sh, tn, dm, mf, dv, kw]	44,196
2	exp thorax pain/di [Diagnosis]	2,481
3	exp thorax pain/	36,580
4	1 or 3	63,756
5	Coronary artery disease or cardiac disease or coronary heart disease or coronary thrombosis or coronary occlusion).mp. [mp = ti, ab, hw, tc, id, ot, tm, sh, tn, dm, mf, dv, kw]	222,468
6	4 not 5	55,961
7	(sensitivity or specificity or diagnostic tests).mp. [mp = ti, ab, hw, tc, id, ot, tm, sh, tn, dm, mf, dv, kw]	1,162,714
8	2 or 7	1,164,908
9	6 and 8	4,565
10	Limit 9 to human	4,180
11	Limit 10 to year = ‘1992-Current’	3,778
12	Limit 11 to ‘300 adulthood < age 18 years and older > ‘ [Limit not valid in Embase; records were retained]	3,769
13	Limit 12 to adulthood <18+ years > [Limit not valid in Embase; records were retained]	3,769

## Appendix 2: Set up of the hierarchical Bayesian models for the summary receiver operating characteristic (ROC) curves

Model 1: proton pump inhibitor (PPI) studies

Assumption: imperfect reference standard

Prior distributions:

Prior of prevalence (pi) is beta (12, 12), <= > pi in [0.3, 0.7]

Prior of beta is uniform (−0.75, 0.75)

Prior of THETA is uniform (−1.5, 1.5)

Prior of LAMBDA is uniform (−3, 3)

Prior of sigma_alpha is uniform (0, 2)

Prior of sigma_theta is uniform (0, 2)

Prior of S2 (sensitivity of reference test) is:

Study(ies) 1 to 7 beta (172.55, 30.45), <= > S2 in [0.8, 0.9]

Prior of C2 (specificity of reference test) is:

Study(ies) 1 to 7 beta (50.4, 12.6), <= > C2 in [0.7, 0.9]

Model 2*:* exertional 24 h pH-metry

Assumption: imperfect reference standard

Prior distributions:

Prior of prevalence (pi) is beta (5.2318, 6.0194), <= > pi in [0.18, 0.75]

Prior of beta is uniform (−0.75, 0.75)

Prior of THETA is uniform (−1.5, 1.5)

Prior of LAMBDA is uniform (−3, 3)

Prior of sigma_alpha is uniform (0, 2)

Prior of sigma_theta is uniform (0, 2)

Prior of S2 (sensitivity of reference test) is:

Study(ies) 1 to 5 beta (172.55, 30.45), <= > S2 in [0.8, 0.9]

Prior of C2 (specificity of reference test) is:

Study(ies) 1 to 5 beta (50.4, 12.6), <= > C2 in [0.7, 0.9]

## Appendix 3: Summary of excluded studies during full-text review

In Table 5 summarizes the studies reviewed in full-text and excluded from the systematic review. For each study the reason for exclusion is provided.

**Table 5:** Summary of excluded studies during full-text review

**Author**	**Year**	**Design**	**Comments**
Aanen	2008	cohort, prospective	No diagnostic study. No NCCP. GERD, reproducibility of reflux symptoms only
Abbass	2009	randomised clinical trial	No diagnostic study. No NCCP. General pain patients
Achem	1993	retrospective, review	Prevalence of nutcracker esophagus in NCCP. For treatment outcome open label trial with small sample
Achem	1997	randomised, controlled trial	No diagnostic study. GERD patients only received PPI
Achem	2000	review article	Review article about atypical chest pain
Adams	2001	retrospective review	no NCCP. Spiral CT in pulmonary embolism
Adamek	1995	cross-sectional study	No reference test. Description of coexistence of motility disorders and pathologic acid reflux
Aikens	2001	cross-sectional study	No diagnostic study, presence of fear in NCCP patients. Correlation of fear with symptoms
Aizawa	1993	cross-sectional study	no NCCP, acetylcholine provocation test for coronary arterial spasm
Ajanovic	1999	cross-sectional study	no NCCP. Pulmonary embolism
Aksglaede	2003	experimental	Experimental. Small sample (n = 5) chest pain.
Alexander	1994	cross-sectional study	Prevalence and nature of mental disorders in NCCP and IHD
Amarasiri	2010	cross-sectional study	GERD patients not NCCP
Anzai	2000	cross-sectional study	No reference test, coronary flow reserve with dopler in patients with no significant coronary stenosis
Armstrong	1992	review article	Review article about atypical chest pain
Arnold	2009	randomised clinical trial	No diagnostic study. Treatment outcome
Aufderheide	1996	validation	Validation of ACI-TIPI probabilities for MI
Bak	1994	cohort, prospective	No diagnostic study. Comparison of prevalence of findings
Balaban	1999	experimental	No diagnostic study. Small sample (n = 10)
Baniukiewicz	1997	cross-sectional study	No diagnostic study. Description of findings in upper GI studies
Barham	1997	observational	No NCCP. Description of presence of esophageal spasm in patients undergoing upper GI studies
Barki	1996	cohort, prospective	No diagnostic study. Description of clinical presentation in painful rip syndrome
Basseri	2011	experimental	Experimental. No NCCP. Different techniques swallow studies
Bassotti	1998	cohort, retrospective	No NCCP. Nutcracker esophagus and the symptoms and findings investigated.
Bassotti	1992	cohort, prospective	No diagnostic study. Prevalence
Beck	1992	cross-sectional study	No diagnostic study. Charasteristics of NCCP patients compared to general pain patients
Belleville	2010	cross-sectional study	No diagnostic study. Characteristics of patients with panic disorders in the ER
Berkovich	2000	cohort, retrospective	No NCCP
Bernstein	2002	validation	GOLDmineR: improvement of a risk model
Berthelot	2005	cohort, retrospective	No diagnostic study. Pain referral study after injection
Bjorksten	1999	cross-sectional study	No patients, workers with muscoloskeletal complaints
Blatchford	1999	cross-sectional study	No NCCP. Emergency medical admission rates
Borjesson	1998	cross-sectional study	Small sample (n = 18), prevalence of esophageal findings
Borjesson	1998	non-randomised controlled trial	No diagnostic study. Small sample size (n = 20 per group). Intervention = TENS
Bortolotti	2001	experimental	No diagnostic study, small sample (n = 9)
Bortolotti	1997	randomised clinical trial	No diagnostic study. L-Arginine in patients with NCCP. Small sample (n = 8)
Bovero	1993	cohort, prospective	Duplicate of same study Bovero 1993 included in the analysis under different titel
Bovero	1993	cohort, prospective	Duplicate of same study Bovero 1993 included in the analysis under different titel
Brims	2010	review article	Review article about atypical chest pain
Broekaert	2006	experimental	Experimental trial in volunteers (no patients, n = 10)
Brunse	2010	cohort, prospective	No diagnostic study. Prevalence
Brusori	2001	cross-sectional study	Mixed sample, diagnosis of esophageal dysmotility in fluoroscopy vs. Manometry
Budzynski	2010	cross-sectional study	Mixed patients sample with significant and non significant coronary leasons not responding to PPI treatment
Bruyninckx	2009	cross-sectional study	No diagnostic study. GP's reasons for referral
Cameron	2006	case series	Case series, selected sample by gastroenterologist. Not all patients had all investigation. Small samples for each group
Cannon	1994	randomised clinical trial	Treatment outcome (imipramine vs. placebo)
Carter	1997	review article	Review article about atypical chest pain
Cremonini	2005	review article	Systematic Review PPI
Castell	1998	Editorial	Editorial
Chambers	1998	observational	Small sample size: n = 23, SI in 7 patients not calculated
Cheung	2007	cross-sectional study	No diagnostic study. Questionnaire to doctors to see what kind of patients they see, what diagnostic tests they use and how they treat.
Christenson	2004	cohort, prospective	Chest discomfort inappropriately not diagnosed ACS. Different research question
Crichton	1997	experimental	Experimental statistical rule out
Cossentino	2012	randomised clinical trial	No diagnostic study. Baclofen in gastro-esophageal diseases
Dekel	2003	cohort, retrospective	No diagnostic study. Prevalence of esophageal motility disorders.
Dekel	2004	Not randomised, not controlled trial	PPI trial only 14 patients included (only GERD positive treated)
Deng	2009	cross-sectional study	No NCCP. Combination between cardiac ischemia and esophageal spasms
De Vries	2006	cross-sectional study	Mixed patient sample with cardiac and non-cardiac chest pain
Dickman	2007	cohort, prospective	No diagnostic study. Prevalence of GI findings in NCCP vs. patients with GERD
Disla	1994	cohort, prospective	No diagnostic study, prevalence
Domanovits	2002	cross-sectional study	Rule out cardiovascular disease. No diagnostic study for NCCP
Ellis	1992	cohort, prospective	No diagnostic study. Treatment outcome in patients with esophageal spasm
Elloway	1992	cross-sectional study	provocative radionuclide esophagealNo comparison to reference test, radionuclide esophageal transit (P-RET) investigation, small sample (n = 30)
Elloway	1992	cross-sectional study	Same study under different title
Erhardt	2002	Guideline	Task force on the management of chest pain
Esayag	2008	retrospective review	Pleuritic chest pain. No reference test, description of presentation and outcome
Esler	2001	randomized clinical trial	Dissertation, same as following.
Esler	2003	randomized clinical trial	Treatment intervention in NCCP. CBT in NCCP seems to reduce chest pain episodes
Fass	1999	cohort, prospective	No diagnostic study. Treatment outcome study
Fleischmann	1997	cross-sectional study	No NCCP. Echokardiographic findings in acute chest pain and health status
Fletcher	2011	cohort, prospective	Sample size: 8 patients with NCCP
Fornari	2008	cohort, retrospective	No NCCP. Only nutcracker esophagus investigated
Fournier	1993	cross-sectional study	No NCCP. Ergovine test during coronary angiogram and induction of coronary spasm
Fournier	1993	cross-sectional study	Same study under different titles
Foldes	2011	cross-sectional study	No comparison of test with reference test. Prevalence of panic disorders
Frobert	1996	cross-sectional study	No diagnostic study. Comparison of characteristics between NCCP with positive treadmill test compared to negative treadmill test.
Gentile	2003	cross-sectional study	Patients with pneumococcal pneumonia. No reference test, description of presentation, microbiological findings and mortality
Gignoux	1993	experimental	No diagnostic study, experimental study
Goehler	2011	experimental	Simulation model of CT scan
Goodacre	2004	randomized clinical trial	No diagnostic study. Comparison of treatments
Gustafsson	1997	non-randomised controlled trial	Sample size: intravenous edrophonium chloride test in 16 patients
Ha	1998	cross-sectional study	No only NCCP. Patients with suspected coronary artery spasm. Ergonovine provocation test and scintigrafic findings. Small sample (n = 26)
Hamm	2011	Guideline	ESC Guidelines
Herbella	2009	cross-sectional study	No NCCP. Presentation of GERD patients
Hess	2008	validation	Diagnostic accuracy to exclude coronary artery disease
Hillis	2003	observational	Correlation of predictors and long term outcome. No diagnostic study
Hick	1992	cohort, retrospective	No diagnostic study. Comparison of characteristics
Hirano	2001	cross-sectional study	No NCCP. Coronary artery spasm
Ho	2001	cross-sectional study	No diagnostic study. Difference between cardiovascular disease and non-cardiovascular disease
Hobson	2006	experimental	Experimental for pain threshold.
Hobson	2006	experimental	Same study under different titels
Howarth	2003	cohort, prospective	Ischemic heart disease and GERD
Hu	2000	experimental	Experimental trial in volunteers
Hughes	2007	cohort, retrospective	No diagnostic study. Risk factors for Reflux or NCCP
Hung	2010	review article	Review article about atypical chest pain
Ilgen	2011	validation	Diagnostic accuracy to exclude coronary artery disease
Jacobs	2007	Guideline	executive summary of management patients with ischemic heart disease
Jerlock	2005	qualitative study	No diagnostic study. Qualitative study
Johnston	1993	cohort, retrospective	No diagnostic study, prevalence
Jones	1999	cross-sectional study	Pleuritic chest pain. Review article
Kahrilas	2011	systematic review	Systematic Review PPI Trial in NCCP, reference publication for included studies
Kao	1993	cross-sectional study	No diagnostic study, prevalence
Karamanolis	2008	experimental	Healthy volunteers
Karlson	1994	observational	No diagnostic study. Prognosis and outcome after discharge for ER
Ke	1993	cohort, prospective	No diagnostic study, prevalence of GERD in NCCP
Keefe	2011	randomised clinical trial	No diagnostic study. Coping skills training, sertraline, placebo
Keogh	2004	cross-sectional study	No diagnostic study. Presence of various psychological factors in the ER in cardiac vs. NCCP on cardiac chest pain
Kisley	1997	cohort, prospective	No diagnostic study. Prognosis after discharge after first admission with acute chest pain
Klingerman	2011	cross-sectional study	Rule out cardiovascular disease. No diagnostic study for NCCP
Klopocka	2005	cohort, prospective	No diagnostic study. Only 24 patients with NCCP
Klopocka	2005	cohort, prospective	Same study under different titles
Koop	2005	journal article	GERD. No diagnostic study in NCCP
Kumarathurai	2008	cohort, prospective	No diagnostic study.
Kushner	1992	cross-sectional study	small sample: n = 27. Presence of panic disorders in relatives.
Lacy	2009	cross-sectional study	Prevalence study
Lam	1994	cohort, prospective	No diagnostic procedure. Only patients included that chest pain was reproduced during the investigation
Lanzarini	1994	cross-sectional study	Description of findings in dobutamine stress echocardiography in patients with positive exercise stress test and negative coronar angiography including ergonovine stress test.
Lauenbjerg	1997	observational	No diagnostic study. Long term prognosis in patients with NCCP of various etiologies
Lee	2011	randomised clinical trial	Conference proceeding. No diagnostic study
Lee	2005	cross-sectional study	Prevalence study
Lehtola	2010	randomised, controlled trial	Treatment outcome (manipulation, acupuncture vs. placebo. No diagnostic study
Lessard	2012		Patients with NCCP with Panic disorders. Two different interventions, no diagnostic study
Lien	2011	cohort, prospective	NCCP not investigated
Lin	2004	cross-sectional study	No NCCP. GERD symptoms and underlying conditions. Differences between women and men
Liu	2006	cross-sectional study	No NCCP patients. Correlation analysis between psychological factors and findings
Lopez Gaston	1994	cross-sectional study	Only esophageal pain investigated not NCCP
Lopez Gaston	1994	cross-sectional study	Same study under different titles
Lopez Gaston	1994	cross-sectional study	Same study under different titles
Loten	2009	observational	No diagnostic study. Adverse outcome / prognosis of mixed patient population
Lyer	2009	retrospective review	Spontaneous pneumomediastinum. Presentation and findings. No diagnostic study
MacPherson	2007	cross-sectional study	No diagnostic study. Survey of patients after ER diagnosis NCCP about interest in acupuncture
Maev	2007	randomised clinical trial	Conference proceeding
Maev	2007	randomised clinical trial	Same study under different titles
Makk	2000	experimental	Experimental study. No diagnostic study. Comparison of acid infusion and cardiac vs. non-cardiac chest pain. Small sample
Manchikanti	2003	observational	No NCCP. Medial branch blocks for musculoskeletal pain
Manterola	2004	cross-sectional study	No diagnostic study. Clinical presentation of patients with NCCP
Martina	1997	cross-sectional study	All patients presenting in primary care. NCCP not as subgroup investigated
Matthews	2005	Meta-analysis	Meta-analysis for PPI-Trial
Mayou	1994	cohort, prospective	No diagnostic study. Comparison of NCCP vs. IHD patients
Mayou	2002	cohort, prospective	No diagnostic study. One year follow-up in comparison to cardiac chest pain. Comparison of costs to the CVD patients
Mearin	1998	cohort study, prospective	No diagnostic study. Change of habits during manometry
Mehta	1995	experimental	No reference test as “true positive” defined.
Mendelson	1997	cohort, prospective	Reference test are cancer patients. Comparison of Szintigraphy for the diagnosis of costochondritis
Mitchell	2006	cross-sectional study	Prevalence of risk factors and pretest probability
Miniati	1999	cross-sectional study	no NCCP. Pulmonary embolism
Miniati	2001	cross-sectional study	no NCCP. Pulmonary embolism
Miniati	2003	cross-sectional study	no NCCP. Prediction model for pulmonary embolism
Mujica	2001	cohort, prospective	No diagnostic study in patients. Healthy volunteers
Mulero	1999	cross-sectional study	Small sample (n = 24). No reference test. Descriptive findings in SPECT
Munk	2008	cohort, prospective	No diagnostic study. Risk of death in patients with unexplained chest pain
Nanbu	1997	cross-sectional study	Focus on difference between IHD and NCCP patients. Valdity of the medical interview for patients with NCCP
Nasr	2010	experimental	Experimental study.
Nasr	2010	experimental	Same study under different titels
Nellemann	2000	experimental	Small sample (n = 5 with chest pain)
Nevitt	1999	secondary analysis of an RCT	No NCCP. Vertebral fracture
Nikolic	2010	cross-sectional study	No diagnostic study. Comparison of NCCP to IHD patients
Nilsson	2003	cross-sectional study	Prevalence of Diagnosis of IHD in primary care.
Okada	1993	cross-sectional study	No NCCP. Provocation of myocardial ischemia by hyperventilation
Oliver	1999	cross-sectional study	CT in acute non-cardiac chest pain. No reference. Description of diagnoses found.
Pandak	2002	cross-sectional study	duplicate of the study included in the analysis
Panju	1996	observational	No diagnostic study. Patients prognosis after discharge
Paterson	1993	cohort, prospective	exclude, small sample
Paterson	1995	cross-sectional study	No diagnostic study. Description of finding in balloon distention NCCP in comparison to other pain patients
Paterson	1996	cross-sectional study	Not enough information to populate a two by two table. Small sample (n = 23)
Porter-Moffitt	2006	cross-sectional study	Small sample (Chest pain n = 34). Comparison of findings to other pain diagnosis.
Rasmussen	2009	cohort, prospective	No NCCP. Complex regional pain syndrome
Robertson	2008	cross-sectional study	Comparison of psychological morbidity in cardiac vs. Non-cardiac chest pain
Rosano	1996	randomised clinical trial	No diagnostic study. Treatment of 17-beta-estradiol patches compared to placebo on NCCP in postmenopausal women
Ratnaike	1993	retrospective review	No diagnostic study. Audit for IHD
Rate	1999	experimental	healthy volunteers
Rencoret	2006	cross-sectional study	No diagnostic study. Prevalence of esophageal disorders in patients with NCCP compared to other GI diseases
Repasky	2005	validation	ED chest pain pathway
Rokkas	1992	cross-sectional study	Not enough information to populate the two by two table
Rose	1994	cohort, prospective	No diagnostic study. Does esophageal testing prevent persistence of symptoms? No control group
Rose	1994	cohort, prospective	Same study under different titles
Rosengren	2008	Editorial	Editorial
Rousset	2011	retrospective review	No NCCP. Catammenial pneumothorax and endometriosis-related pneumothorax
Ruigomez	2004	cohort study	No diagnostic study. Description of risk factors, incidence and comorbidities
Sakata	1996	cross-sectional study	No reference test. Description of homeostasis and fibrinolysis in patients with coronary artery spasm. Not sure only NCCP patients
Sakamoto	2011	cross-sectional study	Acute chest pain, rule out aortic dissection or pulmonary embolism
Salles	2011	cohort, retrospective	No diagnostic study: SAPHO syndrome, clinical characteristics.
Sanchis	2008	cross-sectional study	clinical risk profile of patients with acute chest pain without ST-segment deviation or troponin elevation
Scarinci	1994	cohort, prospective	No NCCP. Women with GERD clinical presentation
Schima	1992	cohort, prospective	Small sample (n = 4 NCCP)
Schima	1992	cohort, prospective	Same study under different titles
Schmidt	2002	cross-sectional study	No NCCP. General pain patients
Schmulson	2004	review article	Review article about atypical chest pain
Shahid	2005	cross-sectional study	No diagnostic study. Prensentation of young adults with chest pain
Shapiro	2006		No NCCP patients. Functional heart burn (pH Man normal) vs. NERD (ph manometry pathologic) incl. psychometric profile).
Sharma	2010	experimental	healthy volunteers
Shelby	2009	randomized clinical trial	Same sample as 210 and 217. Description of psychological factors at baseline.
Sigurdsson	2009	retrospective review	Retrospective analysis of lung biopsy. No NCCP group
Singh	1993	retrospective review	duplicate of the included study
Smith	2000	retrospective review	Feasibility study based on records in a chiropractic clinic.
Smout	1992	experimental	experimental. Small sample (n = 10)
Sobralske	2005	review article	Review article about atypical chest pain
Spencer	2006	observational	No NCCP. GI patients long term outcome
Sporer	2007	cross-sectional study	Rule out cardiovascular disease. No diagnostic study for NCCP
Stahl	1994	cohort, prospective	Small sample (n = 13 NCCP patients)
Steurer	2010	Metaanalysis	clinical value of diagnostic instruments to rule out IHD
Stochkendahl	2008	randomised clinical trial	Study protocol
Stochkendahl	2012	randomised, controlled trial	No diagnostic study. Treatment outcome
Stochkendahl	2012	randomised, controlled trial	No diagnostic study. Same study as previous. Treatment outcome 1 year follow-up
Stollman	1997	cross-sectional study	Small sample (n = 14 patients)
Taylor	2002	cross-sectional study	Rule out strategy cardiovascular patients. No diagnostic study in NCCP
Taniguchi	2009	cross-sectional study	Chest pain in asthma. Treatment response to bronchodilatators
Tew	1995	cross-sectional study	No diagnostic study. Outcome cardiovascular patients
Tougas	2001	experimental	Experimental. Autonomic reaction to acid infusion in NCCP patients (n = 28) compared to controls
Triadafilopoulos	1997	cohort, prospective	GERD patients. Description of spectrum of patients. Only a few with NCCP
Tutuian	2006	cross-sectional study	Not enough information to populate a two by two table in NCCP patients with esophageal spasm
Valdovinos	2004	experimental	No reference test. pH Bravo- capsule. Safety, efficacy and experience in 11 patients
Van Kleef	1995	observational	No diagnostic study. Intervention success comparison after radiofrequency lesion of the dorsal root ganglion
Van Peski-Oosterbaan	1998	cross-sectional study	Survey about how people are interested in psychological treatment after discharge after cardiac unit admission. Mixed patients sample.
van Ravensteijn	2012	systematic review	Diagnostic test efficacy in various pain patients
Varia	2000	randomised, controlled trial	No diagnostic study. Comparison efficacy Sertraline vs. Placebo.
Vent	2010	journal article	dysphagia cause of chest pain
Verdon	2010	observational	Chest pain early diagnostic guess accuracy in GP’s
Vermeltfoort	2009	cross-sectional study	Mixed patient sample with cardiac and non-cardiac chest pain
Volpicelli	2008	cohort, prospective	Pleural sonography for the diagnosis of pulmonary embolism
Wang	2011	cross-sectional study	Conference proceeding
Watkins	2011	journal article	Diagnosis and management of community-acquired pneumonia
Weiner	2006	journal article	Cardiac markers in low-risk patients. No study
Weingarten	1993	cross-sectional study	No diagnostic study for NCCP. Reduction of length of stay by complying the guidelines
White	2011	cross-sectional study	no diagnostic study. Prevalence
Wong	2002	cohort, prospective	no comparison to a reference test. Descriptive information only.
Wulsin	2002	randomised clinical trial	no diagnostic study. Treatment of paroxetine vs. usual care
Yelland	2010	review article	Review article about atypical chest pain
Yu	1997	cross-sectional study	Not enough information to populate a two by two table for symptom index and the presence of GERD
Zalar	1995	cross-sectional study	Conference proceeding
Zarauza	2003	observational	No diagnostic study. Follow-up after discharge
Zheng	2008	cross-sectional study	Small sample (n = 27)
Zheng	2008	cross-sectional study	Same study under different title

## Appendix 4: Summary of the Scottish Intercollegiate Guidelines Network (SIGN) quality assessment [19]

In Table 6 the study quality assessed by using the Scottish Intercollegiate Guidelines Network (SIGN) methodology checklist for diagnostic studies [19] is summarized. Study quality was assessed by two reviewers independently.

**Table 6:** Summary of the Scottish Intercollegiate Guidelines Network (SIGN) quality assessment [19]

**Lead author/study**	**1.1**	**1.2**	**1.3**	**1.4**	**1.5**	**1.6**	**1.7**	**1.8**	**1.9**	**1.10**	**1.11**	**1.12**	**1.13**	**2.1**
Dickman [31]	WC	WC	WC	WC	WC	WC	WC	WC	WC	WC	WC	WC	N/A	++
Bautista [32]	WC	WC	WC	WC	WC	WC	WC	WC	WC	WC	WC	WC	N/A	++
Fass [33]	WC	WC	WC	WC	WC	WC	WC	WC	WC	WC	WC	WC	WC	++
Pandak [34]	WC	WC	WC	WC	WC	WC	WC	WC	WC	WC	WC	PA	PA	+
Kim [35]	WC	WC	WC	WC	WC	WC	WC	WC	WC	WC	WC	PA	PA	++
Xia [36]	WC	WC	WC	WC	WC	WC	WC	WC	WC	WC	WC	WC	WC	++
Kushinir [37]	WC	WC	AA	PA	WC	WC	WC	WC	WC	NA	NA	N/A	PA	+
Lacima [38]	WC	PA	WC	WC	WC	WC	WC	WC	WC	NA	NA	WC	WC	+
Cooke [39]	WC	AA	WC	WC	WC	WC	WC	WC	WC	NA	NA	WC	WC	+
Bovero [40]	WC	PA	WC	WC	WC	WC	WC	WC	WC	NA	NA	WC	WC	+
Romand [41]	WC	AA	WC	WC	WC	WC	WC	WC	WC	NA	NA	WC	WC	+
Abrahao [42]	WC	WC	WC	WC	WC	WC	WC	WC	WC	NA	NA	WC	WC	++
Ho [29]	WC	PA	WC	WC	WC	WC	WC	WC	WC	NA	NA	PA	WC	+
Kim [24]	WC	WC	WC	WC	WC	WC	WC	WC	WC	NA	NA	WC	WC	++
Hong [25]	WC	WC	WC	WC	WC	WC	WC	WC	WC	NA	NA	WC	WC	++
Netzer [26]	WC	AA	WC	WC	WC	WC	WC	PA	WC	NA	NA	WC	WC	+
Mousavi [27]	WC	WC	WC	WC	WC	WC	WC	WC	WC	NA	NA	WC	WC	++
Singh [28]	WC	PA	WC	WC	WC	WC	WC	AA	WC	NA	NA	WC	WC	+
Lam [30]	WC	PA	WC	WC	WC	WC	WC	WC	WC	NA	NA	WC	WC	+
Achem [43]	WC	WC	WC	WC	WC	WC	WC	AA	WC	NA	NA	WC	WC	+
Demiryoguran [48]	WC	WC	WC	WC	WC	WC	WC	WC	WC	WC	WC	WC	WC	++
Foldes-Busque [49]	WC	WC	WC	WC	WC	WC	WC	WC	AA	WC	NA	WC	N/A	++
Kujipers [47]	WC	WC	WC	WC	WC	WC	WC	WC	WC	NA	NA	WC	WC	++
Katerndahl [51]	WC	AA	WC	WC	WC	WC	WC	WC	WC	WC	WC	WC	WC	++
Fleet [50]	WC	WC	WC	WC	WC	WC	WC	WC	WC	WC	WC	WC	WC	++
Stochkendahl [44]	WC	WC	WC	NA	WC	WC	PA	WC	WC	NA	NA	WC	WC	+
Manchikanti [46]	WC	WC	WC	WC	PA	PA	WC	WC	WC	WC	PA	PA	WC	+
Bosner [45]	WC	WC	WC	WC	WC	WC	WC	AA	WC	WC	AA	WC	WC	++

1.1: spectrum of patients is representative of patients who will receive the test; 1.2: selection criteria described; 1.3: reference standard is likely to classify the condition correctly; 1.4: period between reference standard and index test short enough; 1.5: whole sample received verification of diagnosis; 1.6: patients receive same reference test regardless of index test results; 1.7: reference standard independent of index test; 1.8: execution of index test described in detail; 1.9: reference standard described in detail; 1.10: index test interpreted without knowledge of result of reference test; 1.11: reference standard results interpreted without knowledge of result index test; 1.12: uninterpretable or intermediate results are reported; 1.13: explanation is provided for withdrawals; 2.1: reliability of the conclusion of the study. Risk of bias (2.1) is as follows. (++), high quality: most of the criteria have been fulfilled. If not fulfilled, the conclusions of the study are very unlikely to alter. (+), moderate quality: some criteria fulfilled. Criteria not adequately described are unlikely to alter the conclusions. (−), low quality: few or no criteria fulfilled. The conclusions are likely to alter.

*AA* adequately addressed, *N/A* not applicable, *NA* not addressed, *NR* not reported, *PA* poorly addressed, *WC* well covered.

## Appendix 5: Summary of all tests evaluated

Table 7 provides a detailed description of all tests and reference tests investigated. Sensitivity, specificity, positive predictive value (PPV), negative predictive value (NPV), prevalence, and post test prevalence are given.

**Table 7:** Summary of all tests evaluated

**Criteria**	**Evaluated test**	**Reference standard**	**TP**	**FP**	**FN**	**TN**	**Sensitivity**	**Specificity**	**PPV**	**NPV**	**LR+**	**LR-**	**Prevalence**	**Post-test + prevalence**	**Post-test - prevalence**
Symptoms															
Kim *et al*. [24]	NCCP with atypical GERD symptoms	Endoscopy (LA classification) and/or 24 h pH-metry (>4%, pH <4	3	20	5	6	0.38	0.23	0.13	0.55	0.49	2.71	24	13	45
Kim *et al*. [24]	NCCP with typical GERD symptoms	Endoscopy (LA classification) and/or 24 h pH-metry (>4%, pH <4	11	2	5	6	0.69	0.75	0.85	0.55	2.75	0.42	67	85	45
Mousavi *et al*. [27]	NCCP with typical GERD symptoms	GERD if two tests positive: endoscopy (Hentzel-Dent), Bernstein test, omeprazole trial	11	5	24	38	0.31	0.88	0.69	0.61	2.70	0.78	45	69	39
Mousavi *et al*. [27]	NCCP relieved by antacid	GERD if two tests positive: endoscopy (Hentzel-Dent), Bernstein test, omeprazole trial	15	36	20	7	0.43	0.16	0.68	0.64	0.51	3.51	45	29	74
Mousavi *et al*. [27]	NCCP and heartburn in history	GERD if two tests positive: endoscopy (Hentzel-Dent), Bernstein test, omeprazole trial	14	8	21	35	0.40	0.81	0.64	0.63	2.15	0.74	45	64	38
Mousavi *et al*. [27]	NCCP and regurgitation in history	GERD if two tests positive: endoscopy (Hentzel-Dent), Bernstein test, omeprazole trial	17	7	18	36	0.49	0.84	0.71	0.67	2.98	0.61	45	71	33
Hong *et al*. [25]	NCCP	Manometry (Specler 2001 criteria) and/or 24 h pH-metry (>4% pH <4)	72	114	128	148	0.36	0.56	0.39	0.54	0.83	1.13	43	39	46
Hong *et al*. [25]	Control: dysphagia	Manometry (Specler 2001 criteria) and/or 24 h pH-metry (>4% pH <4)	27	26	181	228	0.13	0.90	0.51	0.56	1.27	0.97	45	51	44
Hong *et al*. [25]	Control: GERD-typical symptoms	Manometry (Specler 2001 criteria) and/or 24 h pH-metry (>4% pH <4)	53	53	151	205	0.26	0.79	0.50	0.58	1.26	0.93	44	50	42
Hong *et al*. [25]	Dysphagia	Manometry	16	37	84	325	0.16	0.90	0.30	0.80	1.57	0.94	22	30	21
Hong *et al*. [25]	Dysphagia	24 h pH-metry	4	49	63	346	0.06	0.88	0.08	0.85	0.48	1.07	15	8	15
Hong *et al*. [25]	Dysphagia	Manometry and 24 h pH-metry	7	46	23	386	0.23	0.89	0.13	0.94	2.19	0.86	7	13	6
Hong *et al*. [25]	NCCP	Manometry	34	152	63	213	0.35	0.58	0.18	0.77	0.84	1.11	21	18	23
Hong *et al*. [25]	NCCP	24 h pH-metry	29	157	43	233	0.40	0.60	0.16	0.60	1.00	1.00	16	16	16
Hong *et al*. [25]	NCCP	Manometry and 24 h pH-metry	9	177	22	254	0.29	0.59	0.05	0.92	0.71	1.20	7	5	8
Hong *et al*. [25]	GERD-typical symptoms	Manometry	19	87	81	275	0.19	0.76	0.18	0.77	0.79	1.07	22	18	23
Hong *et al*. [25]	GERD-typical symptoms	24 h pH-metry	23	83	49	307	0.32	0.79	0.22	0.86	1.50	0.86	16	22	14
Hong *et al*. [25]	GERD-typical symptoms	Manometry and 24 h pH-metry	11	95	20	336	0.35	0.78	0.10	0.94	1.61	0.83	7	10	6
Netzer *et al*. [26]	NCCP	Manometry and/or 24 h pH-metry (>10.5% pH <4)	31	14	223	35	0.12	0.71	0.69	0.14	0.43	1.23	84	69	86
Netzer *et al*. [26]	Control: GERD-typical symptoms	Manometry and/or 24 h pH-metry (>10.5% pH <4)	127	16	127	33	0.50	0.67	0.89	0.21	1.53	0.74	84	89	79
Netzer *et al*. [26]	Control: dysphagia	Manometry and/or 24 h pH-metry (>10.5% pH <4)	48	8	206	41	0.19	0.84	0.86	0.17	1.16	0.97	84	86	83
Netzer *et al*. [26]	GERD-typical symptoms	24 h pH-metry	115	28	49	111	0.70	0.80	0.80	0.69	3.48	0.37	54	80	31
Netzer *et al*. [26]	Dysphagia	24 h pH-metry	6	50	158	89	0.04	0.64	0.11	0.36	0.10	1.50	54	11	64
Netzer *et al*. [26]	NCCP	24 h pH-metry	24	21	140	118	0.15	0.85	0.53	0.46	0.97	1.01	54	53	54
PPI trial															
Dickman *et al*. [31]	Rabeprazole 20 mg twice a day for 1 week SIS ≥50%	Endoscopy (Hentzel-Dent grades) and/or 24 h pH-metry (>4.2% pH <4)	12	2	4	17	0.75	0.89	0.86	0.81	7.13	0.28	46	86	19
Dickman *et al*. [31]	Placebo for 1 week	Endoscopy (Hentzel-Dent grades) and/or 24 h pH-metry (>4.2% pH <4)	3	4	13	15	0.19	0.79	0.43	0.54	0.89	1.03	46	43	46
Bautista *et al*. [32]	Lansoprazole 60 mg AM, 30 mg PM for 1 week SIS ≥50%	Endoscopy (Hentzel-Dent grades) and/or 24 h pH-metry (>4.2% pH <4)	14	2	4	20	0.78	0.91	0.875	0.833	8.56	0.24	45	88	17
Bautista *et al*. [32]	Lansoprazole 60 mg AM, 30 mg PM for 1 week SIS ≥65%	Endoscopy (Hentzel-Dent grades) and/or 24 h pH-metry (>4.2% pH <4)	15	1	3	21	0.83	0.95	0.93	0.88	18.33	0.17	45	94	13
Bautista *et al*. [32]	Placebo for 1 week	Endoscopy (Hentzel-Dent grades) and/or 24 h pH-metry (>4.2% pH <4)	4	8	14	14	0.22	0.64	0.33	0.50	0.61	1.22	45	33	50
Fass *et al*. [33]	Omeprazole 40 mg AM, 20 mg PM for 1 week SIS ≥50%	Endoscopy (Hentzel-Dent grades) and/or 24 h pH-metry (>4.2% pH <4)	18	2	5	12	0.78	0.86	0.90	0.71	5.48	0.25	62	90	29
Fass *et al*. [33]	Placebo for 1 week	Endoscopy (Hentzel-Dent grades) and/or 24 h pH-metry (>4.2% pH <4)	5	1	18	13	0.22	0.93	0.83	0.42	3.04	0.84	62	83	58
Pandak *et al*. [34]	Omeprazole 40 mg twice a day for 2 weeks SIS ≥50%	Endoscopy and/or 24 h pH-metry (>4.2% pH <4)	18	6	2	12	0.90	0.67	0.75	0.86	2.70	0.15	53	75	14
Pandak *et al*. [34]	Placebo for 2 weeks SIS ≥50%	Endoscopy and/or 24 h pH-metry (>4.2% pH <4)	1	3	19	15	0.05	0.83	0.25	0.44	0.30	1.14	53	25	56
Kim *et al*. [35]	NCCP GERD-related (TP) vs NCCP non-GERD-related (TN): rabeprazole for 1 week SIS ≥50%	Endoscopy (LA classification) and/or 24 h pH-metry (>4.0 pH <4)	8	6	8	20	0.50	0.77	0.57	0.71	2.17	0.65	38	57	29
Kim *et al*. [35]	NCCP GERD-related (TP) vs NCCP non-GERD-related (TN): rabeprazole for 2 weeks SIS ≥50%	Endoscopy (LA classification) and/or 24 h pH-metry (>4.0 pH <4)	13	7	3	19	0.81	0.73	0.65	0.86	3.02	0.26	38	65	14
Xia *et al*. [36]	Lansoprazole 30 mg once a day for 4 weeks SIS ≥50%	24 h pH-metry (De Meester pH <4, 7.5 s)	11	8	1	16	0.92	0.67	0.58	0.94	2.75	0.13	33	58	6
Xia *et al*. [36]	Placebo for 4 weeks SIS ≥50%	24 h pH-metry (De Meester pH <4, 7.5 s)	4	7	8	13	0.33	0.65	0.36	0.62	0.95	1.03	38	36	38
Kushnir *et al*. [37]	High-degree response on PPI (not specified)	24 pH-metry (≥4%, pH <4)	40	18	12	28	0.77	0.61	0.69	0.70	1.97	0.38	53	69	30
Kushnir *et al*. [37]	High-degree response on PPI	Positive Ghillibert probability estimate (GPE)	21	37	5	35	0.81	0.49	0.36	0.88	1.57	0.40	27	36	12
Kushnir *et al*. [37]	High-degree response on PPI	Association of chest pain with pH <4 in reference standard: SI ≥50%	19	39	6	34	0.76	0.47	0.33	0.85	1.42	0.52	26	33	15
Kushnir *et al*. [37]	High-degree response on PPI	24 h pH-metry and positive GPE	15	43	2	38	0.88	0.47	0.26	0.95	1.66	0.25	17	26	5
Kushnir *et al*. [37]	High-degree response on PPI	24 h pH-metry and SI ≥50%	16	42	2	38	0.89	0.48	0.28	0.95	1.69	0.23	18	28	5
Kushnir *et al*. [37]	High-degree response on PPI	24 h pH-metry and SI ≥50% and positive GPE	14	44	1	39	0.93	0.47	0.24	0.98	1.76	0.14	15	24	2
Symptom index															
Singh *et al*. [28]	Association of chest pain with pH <4 in reference standard: SI ≥50%	Endoscopy and/or 24 h pH-metry (De Meester >5.5% pH <4)	19	38	15	81	0.56	0.68	0.33	0.84	1.75	0.65	22	33	16
Singh *et al*. [28]	Association of chest pain with pH <4 in reference standard: SI ≥25%	Endoscopy and/or 24 h pH-metry (De Meester >5.5% pH <4)	23	59	11	60	0.68	0.50	0.28	0.85	1.36	0.64	22	28	15
Singh *et al*. [28]	Association of chest pain with pH <4 in reference standard: SI ≥75%	Endoscopy and/or 24 h pH-metry (De Meester >5.5% pH <4)	8	5	26	114	0.24	0.96	0.62	0.81	5.60	0.80	22	62	19
Singh *et al*. [28]	Association of heartburn with pH <4 in reference standard: Symptom Index (SI) ≥50%	Endoscopy and/or 24 h pH-metry (De Meester >5.5% pH <4)	40	32	3	78	0.93	0.71	0.56	0.96	3.20	0.10	28	56	4
Singh *et al*. [28]	Association of heartburn with pH <4 in reference standard: SI ≥25%	Endoscopy and/or 24 h pH-metry (De Meester >5.5% pH <4)	41	40	2	70	0.95	0.64	0.51	0.97	2.62	0.07	28	51	3
Singh *et al*. [28]	Association of heartburn with pH <4 in reference standard: SI ≥75%	Endoscopy and/or 24 h pH-metry (De Meester >5.5% pH <4)	25	25	9	94	0.74	0.79	0.50	0.91	3.50	0.34	28	58	12
Ho *et al*. [29]	Association of chest pain with pH <4 in reference standard: SI >50%	24 h pH-metry (>4% pH <4, 4 s)	3	9	11	38	0.21	0.81	0.25	0.78	1.12	0.97	23	25	22
Lam *et al*. [30]	Association of chest pain with pH <4 in reference standard: SI ≥75%	24 h pH-metry (execution in acute stage)	13	0	15	13	0.46	1.00	1.00	0.48	13.03	0.54	68	97	54
Others															
Lacima *et al*. [38]	24 h-manometry (pH <4)	Manometry during hospital stay	18	24	18	30	0.50	0.56	0.43	0.56	1.13	0.90	40	43	38
Provocation test															
Cooke *et al*. [39]	NCCP during exertional pH-metry	24 h pH-metry (5.5% pH <4 for 10 s)	4	0	15	31	0.21	1.00	1.00	0.67	14.40	0.79	38	90	33
Cooke *et al*. [39]	Control group: CVD with angina: exertional pH-metry	24 h pH-metry (5.5% pH <4 for 10 s)	1	1	2	12	0.33	0.92	0.50	0.86	4.33	0.72	19	50	14
Bovero *et al*. [40]	NCCP with normal ECG during exertional pH-metry	24 h pH-metry (De Meester criteria: >4.5% pH <4))	17	1	29	20	0.37	0.95	0.94	0.41	7.76	0.66	69	94	59
Bovero *et al*. [40]	NCCP at rest: NCCP with normal ECG during exertional pH-metry	24 h pH-metry (De Meester criteria: >4.5% pH <4))	11	1	23	11	0.32	0.92	0.92	0.32	3.88	0.74	74	92	68
Bovero *et al*. [40]	NCCP exertion/mixed: NCCP with normal ECG during exertional pH-metry	24 h pH-metry (De Meester criteria: >4.5% pH <4))	6	0	6	9	0.50	1.00	1.00	0.60	10.00	0.50	57	93	40
Bovero *et al*. [40]	24 h pH-metry (De Meester criteria: >4.5% pH <4))	NCCP with normal ECG during exertional pH-metry	17	29	1	20	0.94	0.41	0.37	0.95	1.60	0.14	27	37	5
Bovero *et al*. [40]	24 h pH-metry (De Meester criteria: >4.5% pH <4))	NCCP at rest: NCCP with normal ECG during exertional pH-metry	11	23	1	11	0.92	0.32	0.32	0.92	1.36	0.26	26	32	8
Romand *et al*. [41]	NCCP: pH <4 for 10 s during exertional pH-metry	24 h pH-metry (De Meester criteria: >4.5% pH <4))	7	14	3	19	0.70	0.58	0.33	0.86	1.65	0.52	23	33	14
Abrahao *et al*. [42]	NCCP reproducible during balloon distension	Endoscopy (Savary-Miller) and/or manometry and/or pH-metry (De Meester criteria: >4.5% pH <4	14	1	21	4	0.40	0.80	0.93	0.16	2.00	0.75	88	93	84
Abrahao *et al*. [42]	NCCP reproducible during Tensilon test	Endoscopy (Savary-Miller) and/or manometry and/or pH-metry (De Meester criteria: >4.5% pH <4	6	2	29	3	0.17	0.60	0.75	0.09	0.43	1.38	88	75	91
Abrahao *et al*. [42]	NCCP reproducible during Bernstein test	Endoscopy (Savary-Miller) and/or manometry and/or pH-metry (De Meester criteria: >4.5% pH <4	9	1	26	4	0.26	0.80	0.90	0.13	1.29	0.93	88	90	87
Abrahao *et al*. [42]	Tensilon and Bernstein Test and balloon distension (+ if 1 test +)	Endoscopy (Savary-Miller) and/or manometry and/or pH-metry (De Meester criteria: >4.5% pH <4	20	3	15	2	0.57	0.40	0.87	0.12	0.95	1.07	88	87	88
Abrahao *et al*. [42]	NCCP reproducible during Tensilon test	Endoscopy (Savary-Miller) and/or pH-metry (De Meester criteria: >4.5% pH <4	6	2	26	6	0.19	0.75	0.75	0.19	0.75	1.08	80	75	81
Abrahao *et al*. [42]	NCCP reproducible during Bernstein test	Endoscopy (Savary-Miller) and/or pH-metry (De Meester criteria: >4.5% pH <4	8	2	24	6	0.25	0.75	0.80	0.20	1.00	1.00	80	80	80
Abrahao *et al*. [42]	NCCP reproducible during balloon distension	Endoscopy (Savary-Miller) and/or pH-metry (De Meester criteria: >4.5% pH <4	13	2	19	6	0.41	0.75	0.87	0.24	1.63	0.79	80	87	76
Abrahao *et al*. [42]	Tensilon and Bernstein Test and balloon distension (+ if 1 test +)	Endoscopy (Savary-Miller) and/or pH-metry (De Meester criteria: >4.5% pH <4	18	5	14	3	0.56	0.38	0.78	0.18	0.90	1.17	80	78	82
Ho *et al*. [29]	NCCP reproducible during Bernstein test	Endoscopy	4	7	3	56	0.57	0.89	0.36	0.95	5.14	0.48	10	36	5
Eosinophilia															
Achem *et al*. [43]	Current GERD symptoms	Esophageal biopsies	10	26	14	121	0.42	0.82	0.28	0.90	2.36	0.71	14	28	10
Achem *et al*. [43]	Male gender or current GERD symptoms	Esophageal biopsies	18	69	6	78	0.75	0.53	0.21	0.93	1.60	0.47	14	21	7
Achem *et al*. [43]	Male gender or any abnormal EoE endoscopic finding	Esophageal biopsies	23	79	1	68	0.96	0.46	0.23	0.99	1.78	0.09	14	23	1
Achem *et al*. [43]	Current GERD symptoms or any abnormal EoE endoscopic finding	Esophageal biopsies	20	63	4	84	0.83	0.57	0.24	0.96	1.94	0.29	14	24	5
Musculoskeletal															
Stochkendahl *et al*. [44]	Biomechanical dysfunction	Standardized examination protocol	112	120	0	70	1.00	0.37	0.48	1.00	1.58	0.00	37	48	0
Stochkendahl *et al*. [44]	≥3 of 5 overall palpation findings	Standardized examination protocol	111	124	1	66	0.99	0.35	0.47	0.99	1.52	0.03	37	47	1
Bosner *et al*. [45]	Chest wall symptom (CWS) score: localized muscle tension, stinging pain, pain reproducible by palpation, absence of cough, cut-off test negative 0 to 1 points	Interdisciplinary consensus: cardiologist, GP, research associate (based on reviewed baseline, follow-up data)	506	318	59	329	0.90	0.51	0.66	0.82	1.82	0.20	47	61	15
Bosner *et al*. [45]	CWS score: localized muscle tension, stinging pain, pain reproducible by palpation, absence of cough, cut-off test negative 0 to 2 points	Interdisciplinary consensus	357	135	208	512	0.63	0.79	0.76	0.67	3.02	0.47	47	72	29
Stochkendahl *et al*. [44]	Anterior chest wall tenderness	Standardized examination protocol	110	134	2	56	0.98	0.29	0.45	0.97	1.39	0.06	37	45	3
Stochkendahl *et al*. [44]	Angina pectoris (uncertain or negative)	Standardized examination protocol	109	147	3	43	0.97	0.23	0.43	0.94	1.26	0.12	37	43	7
Stochkendahl *et al*. [44]	Pain worse on movement of torso	Standardized examination protocol	32	16	80	174	0.29	0.92	0.67	0.69	3.39	0.78	37	67	32
Bosner *et al*. [45]	Pain worse with movement	Interdisciplinary consensus	221	119	344	528	0.39	0.82	0.65	0.61	2.13	0.75	47	65	40
Stochkendahl *et al*. [44]	Positive/possible belief in pain origin from muscle/joints	Standardized examination protocol	108	156	4	34	0.96	0.18	0.41	0.90	1.17	0.20	37	41	11
Stochkendahl *et al*. [44]	Pain relief on pain medication	Standardized examination protocol	25	13	87	177	0.22	0.93	0.66	0.67	3.26	0.83	37	66	33
Bosner *et al*. [45]	Pain reproducible by palpation	Interdisciplinary consensus	351	193	214	454	0.62	0.70	0.68	0.64	2.08	0.54	47	65	32
Stochkendahl *et al*. [44]	Paraspinal tenderness	Standardized examination protocol	90	112	22	78	0.80	0.41	0.45	0.78	1.36	0.48	37	45	22
Bosner *et al*. [45]	Localized muscle tension	Interdisciplinary consensus	346	164	219	483	0.61	0.75	0.71	0.66	2.41	0.52	47	68	32
Stochkendahl *et al*. [44]	Chest pain present now	Standardized examination protocol	92	116	20	74	0.82	0.39	0.44	0.79	1.35	0.46	37	44	21
Bosner *et al*. [45]	Pain now	Interdisciplinary consensus	328	327	237	320	0.58	0.49	0.50	0.57	1.15	0.85	47	50	43
Stochkendahl *et al*. [44]	Pain debut not during a meal	Standardized examination protocol	109	168	3	22	0.97	0.12	0.39	0.88	1.10	0.23	37	39	12
Stochkendahl *et al*. [44]	Sharp pain	Standardized examination protocol	39	35	73	155	0.35	0.82	0.53	0.68	1.89	0.80	37	53	32
Bosner *et al*. [45]	Stinging pain	Interdisciplinary consensus	299	184	266	463	0.53	0.72	0.62	0.63	1.87	0.66	47	62	37
Stochkendahl *et al*. [44]	Hard physical exercise at least once a week	Standardized examination protocol	42	60	70	130	0.38	0.68	0.41	0.65	1.19	0.91	37	41	35
Stochkendahl *et al*. [44]	Pain not provoked during a meal	Standardized examination protocol	109	170	3	20	0.97	0.11	0.39	0.87	1.09	0.25	37	39	13
Stochkendahl *et al*. [44]	Not sudden debut	Standardized examination protocol	53	31	59	159	0.47	0.84	0.63	0.73	2.90	0.63	37	63	27
Bosner *et al*. [45]	Pain >24 h	Interdisciplinary consensus	158	139	407	508	0.28	0.79	0.53	0.56	1.30	0.92	47	54	45
Stochkendahl *et al*. [44]	Age ≤49 years old	Standardized examination protocol	67	54	45	136	0.60	0.72	0.55	0.75	2.10	0.56	37	55	25
Bosner *et al*. [45]	Pain mostly at noon time	Interdisciplinary consensus	13	30	552	617	0.02	0.95	0.31	0.53	0.50	1.02	47	31	48
Bosner *et al*. [45]	Cough	Interdisciplinary consensus	31	129	534	518	0.06	0.80	0.19	0.49	0.28	1.18	47	20	51
Bosner *et al*. [45]	Known IHD	Interdisciplinary consensus	56	122	509	525	0.10	0.81	0.32	0.51	0.52	1.11	47	32	50
Bosner *et al*. [45]	Pain worse with breathing	Interdisciplinary consensus	138	123	427	524	0.24	0.81	0.53	0.55	1.28	0.93	47	53	45
Manchikanti *et al*. [46]	Chronic thoracic pain: lidocaine injection	Bupivacaine injection	22	14	_	_			0.61	_					
Psychiatric															
Kuijpers *et al*. [47]	Anxiety subscale of the Hospital Anxiety and Depression Scale (HADS-A score, cut-off ≥8)	Diagnosis Anxiety disorders (Mini International Neuropsychiatric Interview (gold standard))	195	71	3	75	0.98	0.51	0.73	0.96	2.03	0.03	58	73	4
Demiryoguran *et al*. [48]	Palpitation	Anxiety disorder: HADS-A score (cut-off ≥10)	18	25	31	80	0.37	0.76	0.42	0.72	1.54	0.83	31	41	27
Demiryoguran *et al*. [48]	Fear of dying	Anxiety disorder: HADS-A score (cut-off ≥10)	11	6	38	102	0.22	0.94	0.65	0.73	4.04	0.82	31	65	27
Demiryoguran *et al*. [48]	Light-headedness, dizziness, faintness	Anxiety disorder: HADS-A score (cut-off ≥10)	11	8	38	100	0.22	0.93	0.58	0.73	3.03	0.84	31	58	28
Demiryoguran *et al*. [48]	Chills or hot flushes	Anxiety disorder: HADS-A score (cut-off ≥10)	11	5	38	103	0.22	0.95	0.69	0.73	4.85	0.81	31	69	27
Demiryoguran *et al*. [48]	Shortness of breath	Anxiety disorder: HADS-A score (cut-off ≥10)	13	22	36	86	0.27	0.80	0.37	0.71	1.30	0.92	31	37	29
Demiryoguran *et al*. [48]	Nausea or gastric discomfort	Anxiety disorder: HADS-A score (cut-off ≥10)	9	10	40	98	0.18	0.91	0.47	0.71	1.98	0.90	31	47	29
Demiryoguran *et al*. [48]	Diaphoresis	Anxiety disorder: HADS-A score (cut-off ≥10)	19	12	30	96	0.39	0.89	0.61	0.76	3.49	0.69	31	61	24
Foldes-Busque *et al*. [49]	The Panic Screening Score (derivation population)	Panic disorder diagnosis (structured Anxiety Disorders Interview Schedule for *Diagnostic and Statistical Manual of Mental Disorders*, fourth edition DSM-IV (ADIS-IV))	53	19	31	98	0.63	0.84	0.74	0.76	3.89	0.44	42	74	24
Foldes-Busque *et al*. [49]	The Panic Screening Score (validation population)	Panic disorder diagnosis (structured ADIS-IV)	69	27	61	148	0.53	0.85	0.72	0.71	3.44	0.55	43	72	29
Katerndahl *et al*. [51]	GP diagnosis of panic disorder	Panic disorder (structured clinical Interview of *Diagnostic and Statistical Manual of Mental Disorders*, based on DSM-III-R)	2	2	26	21	0.07	0.91	0.50	0.45	0.82	1.02	55	50	55
Fleet *et al*. [50]	Panic disorder diagnosis: formula including Agoraphobia Cognitions QA, Mobility Inventory for Agoraphobia, Zone 12 Dermatome Pain Map, Sensory McGill Pain QA, Gender, Zone 25 (Validation population)	Panic Disorder (ADIS-R structured interview by psychologist)	32	41	17	122	0.65	0.75	0.44	0.88	2.60	0.46	23	44	12

Sensitivity calculated by TP/(TP + FN); specificity calculated by TN/(FP + TN). Biomechanical dysfunction defined as chest pain presumably caused by mechanical joint and muscle dysfunction related to C4 to T8 somatic structures of the spine and chest wall established by means of joint-play and/or end-play palpation.

24-h pH-metry: 24-h pH monitoring measures with a single sensor located above the lower esophageal sphincter (LES) a reflux event and the association of the reflux event with symptoms can also be ascertained from the tracing; manometry: esophageal manometry measures mean pressure of the lower esophageal sphincter and any degree of hypomotility and dysmotility in the esophagus.

LR+, positive likelihood ratio calculated sensitivity/1 - specificity; LR-, negative likelihood ratio calculated 1 - sensitivity/specificity; diagnostic test accuracy. Very good: LR + >10, LR- <0.1; good: LR + 5 to 10, LR- 0.1 to 0.2; fair: LR + 2 to 5, LR- 0.2 to 0.5; poor: LR + 1 to 2, LR- 0.5 to 1.

Reference tests are as follows. Endoscopic classification: LA classification: grade A, ≥1 mucosal break ≤5 mm, that does not extend between the tops of two mucosal folds; grade B, ≥1 mucosal break >5 mm long that does not extend between the tops of two mucosal folds; grade C, ≥1 mucosal break that is continuous between the tops of two or more mucosal folds but which involves <75% of the circumference; grade D, ≥1 mucosal break which involves at least 75% of the esophageal circumference [52]. Savary-Miller System: grade I, single or isolated erosive lesion(s) affecting only one longitudinal fold; grade II, multiple erosive lesions, non-circumferential, affecting more than one longitudinal fold, with or without confluence; grade III, circumferential erosive lesions; grade IV, chronic lesions: ulcer(s), stricture(s) and/or short esophagus. Alone or associated with lesions of grades 1 to 3; grade V, columnar epithelium in continuity with the Z line, non-circular, star-shaped, or circumferential. Alone or associated with lesions of grades 1 to 4 [53]. Hentzel-Dent grades: grade 0, no mucosal abnormalities; grade 1, no macroscopic lesions but erythema, hyperemia, or mucosal friability; grade 2, superficial erosions involving <10% of mucosal surface of the last 5 cm of esophageal squamous mucosa; grade 3, superficial erosions or ulceration involving 10% to 50% of the mucosal surface of the last 5 cm of esophageal squamous mucosa; grade 4, deep peptide ulceration anywhere in the esophagus or confluent erosion of >50% of the mucosal surface of the last 5 cm of esophageal squamous mucosa [54]. pH-metry: De Meester criteria: (1) total number of reflux episodes; (2) number of reflux episodes with pH <4 for more than 5 minutes; (3) duration of the longest episode; (4) percentage total time pH <4; (5) percentage upright time pH <4; and 6) percentage recumbent time pH <4. [55]. Manometry: Spechler criteria: diagnosis of ineffective esophageal motility, nutcracker esophagus, spasm, achalasia based on: basal lower esophageal sphincter pressure, relaxation, wave progression, distal wave amplitude [56].

*ACQ* Agoraphobia Cognitions Questionnaire, *ADIS-R* structured interview by psychologist, recommended interview protocol for panic research, *CVD* cardiovascular disease, *DSM-IV (ADIS-IV)* Anxiety Disorders Interview Schedule, *GERD* gastroesophageal reflux disease, *GP* general practitioner, *GPE* Ghillibert probability estimate (sum of partial probabilities for exact numbers of reflux associated symptoms within the context of the total number of symptoms), *HDR* high-degree response, *EoE* eosinophilic esophagitis (typical abnormal EoE endoscopic findings (rings or furrows)), *IHD* ischemic heart disease, *McGill sensory* McGill Pain Questionnaire sensory, *MIA* Mobility Inventory for Agoraphobia, *MINI* Mini International Neuropsychiatric Interview (gold standard for anxiety disorders), *NPV* negative predictive value, *PPV* positive predictive value, *SI* symptom index (calculated as the proportion of chest pain symptoms (pH <4) divided by the number of chest pain episodes recorded, expressed as a percentage), *SIS* symptom index score (calculated by adding the reported daily severity (mild = 1; moderate = 2; severe = 3; and disabling = 4) multiplied by the reported daily frequency values during each week).

## Competing interests

The authors declare that they have no competing interests. This study was not funded.

## Authors’ contributions

MW and KR carried out data extraction, participated in the analysis and drafted the manuscript. UH participated in the design of the study and performed the statistical analysis. JS conceived the study, and participated in its design and coordination and helped to draft the manuscript. All authors read and approved the final manuscript.

## Pre-publication history

The pre-publication history for this paper can be accessed here:

http://www.biomedcentral.com/1741-7015/11/239/prepub
